# Complex Dynamics of Virus Spread from Low Infection Multiplicities: Implications for the Spread of Oncolytic Viruses

**DOI:** 10.1371/journal.pcbi.1005241

**Published:** 2017-01-20

**Authors:** Ignacio A. Rodriguez-Brenes, Andrew Hofacre, Hung Fan, Dominik Wodarz

**Affiliations:** 1 Department of Mathematics, University of California, Irvine, Irvine, California, United States of America; 2 Department of Ecology and Evolutionary Biology, University of California, Irvine, Irvine, California, United States of America; 3 Department of Molecular Biology and Biochemistry, Cancer Research Institute, University of California, Irvine, Irvine, California, United States of America; ETH Zurich, SWITZERLAND

## Abstract

While virus growth dynamics have been well-characterized in several infections, data are typically collected once the virus population becomes easily detectable. Earlier dynamics, however, remain less understood. We recently reported unusual early dynamics in an experimental system using adenovirus infection of human embryonic kidney (293) cells. Under identical experimental conditions, inoculation at low infection multiplicities resulted in either robust spread, or in limited spread that eventually stalled, with both outcomes occurring with approximately equal frequencies. The reasons underlying these observations have not been understood. Here, we present further experimental data showing that inhibition of interferon-induced antiviral states in cells results in a significant increase in the percentage of robust infections that are observed, implicating a race between virus replication and the spread of the anti-viral state as a central mechanism. Analysis of a variety of computational models, however, reveals that this alone cannot explain the simultaneous occurrence of both viral growth outcomes under identical conditions, and that additional biological mechanisms have to be invoked to explain the data. One such mechanism is the ability of the virus to overcome the antiviral state through multiple infection of cells. If this is included in the model, two outcomes of viral spread are found to be simultaneously stable, depending on initial conditions. In stochastic versions of such models, the system can go by chance to either state from identical initial conditions, with the relative frequency of the outcomes depending on the strength of the interferon-based anti-viral response, consistent with the experiments. This demonstrates considerable complexity during the early phase of the infection that can influence the ability of a virus to become successfully established. Implications for the initial dynamics of oncolytic virus spread through tumors are discussed.

## Author Summary

We investigate in vitro adenovirus spread starting from the lowest infection multiplicities. This phase of virus dynamics remains poorly understood and is likely critical for ensuring that engineered oncolytic viruses successfully spread and destroy tumors. We find unexpectedly complex dynamics, which are analyzed with a combination of experiments and mathematical models. The experiments indicate that the induction of an interferon-based anti-viral state is a crucial underlying mechanism. The mathematical models demonstrate that this mechanism alone cannot explain the experiments, and that additional mechanisms must be invoked to account for the data. The models suggest that the ability of the virus to overcome the anti-viral state through multiple infection of cells might be one such mechanism.

## Introduction

The dynamics of virus spread have been studied extensively in the context of different infections, both experimentally and with mathematical models [1–3]. In particular, virus growth kinetics have been investigated in vitro and in vivo, in animal models and in human patients (see e.g. [4–14]). From such data, important kinetic parameters have been measured [4,15–19], such as the death rates of infected cells, the rates of viral turnover, and the basic reproductive ratio of the virus, R_0_, which is thought to determine whether a successful infection can be established in a host or not.

Most studies that investigate the spread of a virus through its target cell population, however, only document virus growth once the number of infected cells has already reached relatively large numbers (in part because virus replication is hard to quantify at very low levels of infection). As a consequence, the dynamics during the earliest stages of virus spread remain poorly understood. Yet, this early phase can be crucial in determining the fate of the infection. We have recently studied such early dynamics experimentally in the context of adenovirus spread in vitro [20,21]. We tracked the spread of adenovirus infection in a 2 dimensional monolayer of human embryonic kidney (293) cells. The adenovirus used expressed green fluorescent protein, so that early virus spread from initially infected cells could be followed in space and time. A variety of interesting findings were made. Experiments showed that when virus replication initiated from a single cell, infections failed to take place for a certain fraction of the experiments. However, once at least three infected cells had been generated, a spreading infection was always established [20]. It was hypothesized that in the monolayer culture, multiply infected cells are generated relatively quickly as the number of infected cells increases, and that a high viral production from multiply infected cells could explain the lack of extinction events once three or more infected cells had been generated [20].

Following the spreading virus further (21), two different outcomes were observed: (i) In what can be called a “limited spread” the infected cell population initially increased slowly, but eventually stalled at relatively low infected cell population sizes. (ii) In what can be called "robust spread", the virus infection grew at a much faster pace, did not stall, and eventually reached a large number of infected cells. Importantly, these two outcomes occurred under identical experimental conditions i.e. on the same infected culture dish. In a given culture, a number of infection foci were initiated and followed, and about half of them displayed robust spread, while the other half displayed limited spread. We also note that the limited spread outcomes were not stochastic extinction events caused by random fluctuations around small numbers. Indeed, in a previous study we found that once 3 or more infected cells were generated a spreading infection was always established [20].

The occurrence of two different outcomes of early virus spread under identical experimental conditions was surprising and has remained unexplained. Here, we combine further experiments with mathematical modeling to better understand these dynamics. A relevant biological system in this respect are oncolytic viruses; they specifically replicate in cancer cells and are being explored as a treatment modality [22]. Cancers are typically infected at relatively low multiplicities, with the aim that that the virus spreads throughout the tumor cell population and kills most malignant cells. Promising results have been obtained in clinical trials, some of which involve adenoviruses upon which our experimental system is based [23]. A herpes-based virus has been approved for the treatment of melanomas, but seems to act through induction of immune responses [24]. The potential of the spreading virus itself to consistently control tumors has yet to be realized, and the initial spread from low infection multiplicities appears to be a crucial and limiting phase. Our work helps to shed light onto how this initial barrier can be overcome.

## Results

### Experimental observations

**Fig 1:**
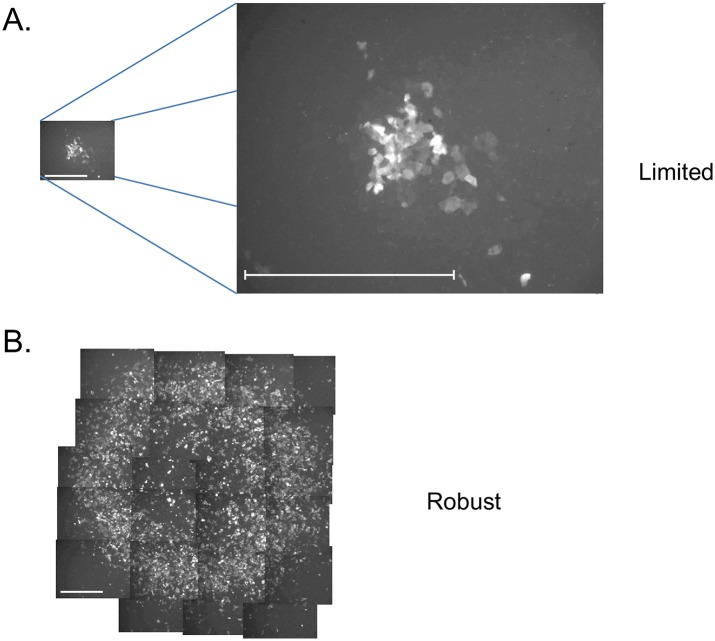
Limited and robust infections. Ad-293 cells were infected with low doses of AdEGFPuci under conditions of plaque formation (10–100 infectious units/5 cm culture dish, agar overlay), and areas of infection were visualized by fluorescence microscopy for GFP. Limited (A) and robust areas (B) were observed on the same plates at 12 days post-infection. The bars represent 500 μ. The limited area of infection is also shown at expanded magnification in panel A. The robust area (B) required multiple photographic fields, and a montage is shown.

The experimental system used in this study has been described previously [20]. Human HEK 293 (Ad-293) cells that express Adenovirus EIA and EIB proteins were infected in monolayer culture with a recombinant Adenovirus expressing jellyfish enhanced green fluorescent protein (EGFP) in place of the EIA and EIB coding region (AdEGFPuci). The infections were carried out with an agar overlay, which restricted viral spread to local cell-cell spread. By conducting the infections on culture dishes with grids, it was possible to repeatedly monitor the same regions of the cultures and count the number of infected (green) cells over time. As described in a previous study, two types of infection outcomes were observed once an initial spreading infection was established—robust spread leading to typical virus plaques, and limited spread where the infection eventually died out (Fig 1). Under the standard infection conditions, the ratio of robust vs. limited infections was approximately 1:1.

**Fig 2:**
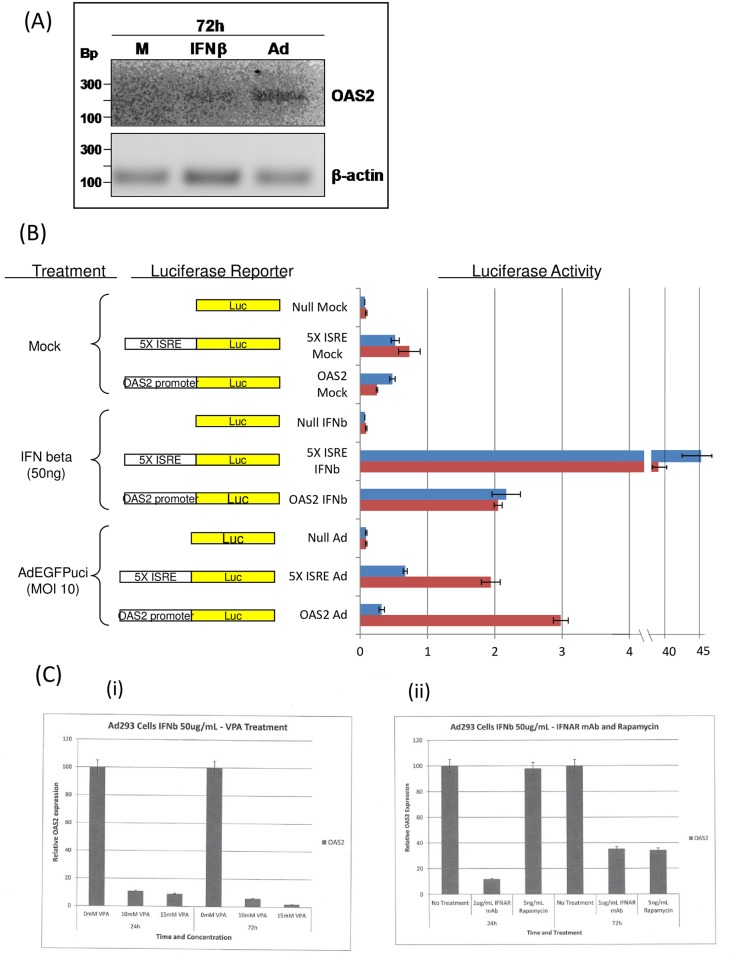
**(A,B) Induction of OAS2 expression by AdEGFPuci infection**. A) Ad-293 cells were infected with AdEGFPuci at an MOI of 10, and RNA was extracted 72 hr post-infection (Ad). For comparison, cells were incubated in medium alone (M or Mock), or they were treated for the same period with 50 μg/ml human interferon β (IFNβ). The RNAs were used in RT-PCR reactions for several interferon-responsive genes; only OAS2 RNA showed significant enhancement after AdEGFPuci infection; low level of enhancement by IFNβ was also observed. Amplification for the same number of cycles for β-actin RNA was performed for normalization. B) Ad-293 cells were transfected with luciferase reporter plasmids driven by a canonical interferon response element (5XISRE), the upstream regulatory sequences of the OAS2 promoter (OAS2), or the equivalent luciferase construct lacking promoter/enhancer sequences (Null). The transfected cells were treated with 50 μg/ml IFNβ, infected with AdEGFPuci (MOI of 10), or not treated (Mock), and lysates from replicate cultures were harvested at 24 and 72 hr. Luciferase assays were carried out using the dual luciferase assay system, and luciferase activities relative to the reference renilla luciferase activity are shown in arbitrary units. Activities at 24 hr are shown in blue, and those at 72 hr are shown in red. Bars indicate standard deviations from replicate cultures. **C) Inhibition of induction of OAS2**. i). Ad-293 cells were incubated with valproic acid (VPA) at different concentrations along with 50 μg/ml IFNβ. At 24 and 72 hr, levels of OAS2 RNA in the cells were measured by qRT-PCR. The levels of OAS2 RNA relative to no VPA treatment (set at 1) are shown for the different VPA concentrations. ii). Ad-293 cells were incubated with 1 μg/ml anti-IFNAR mAb, or 5 ng/ml rapamycin, along with 50 μg/ml IFNβ. qRT-PCR assays for OAS2 RNA (relative to no treatment) are shown for 24 and 72 hr post-treatment. Error bars represent standard deviations of triplicate assays.

As suggested in our previous study one possible explanation for the robust vs. limited viral infections could have been the induction of antiviral responses in the infected cells by interferon [21]. While adenovirus encodes genes that antagonize interferons [25], this is not absolute. We therefore tested if AdEGFPuci infection of Ad-293 cells results in induction of an interferon response. When viruses infect cells, detection of viral infection by cellular sensors leads to production of interferons [25]. The interferons are released from the infected cell where they bind to interferon receptors on the surfaces of the same cell or neighboring uninfected cells; this binding leads to signal transduction and transcriptional activation of a series of IFN-responsive genes (ISGs) and establishment of an antiviral state. Expression of ISGs is indicative of an antiviral state. We tested RNA from AdEGFPuci-infected 293 cells for expression of several ISGs by reverse transcriptase-PCR (RT-PCR). The expression of the ISG oligo-adenylate synthase 2 (OAS2) was consistently induced by AdEGFPuci infection at 72 h, indicative of an antiviral state (Fig 2A). In further experiments we found that the infected cells did not show induction of other ISGs tested, including OAS1, MX-1, IFITM1 and ISG3g, which indicated that the antiviral state induced by AdEGFPuci was partial or relatively weak. We investigated this further by studying transcriptional activation of the OAS2 promoter. 293 cells were transiently transfected with expression plasmids consisting of firefly luciferase driven by either the OAS2 promoter/enhancer, or by an artificial promoter/enhancer containing five tandem copies of the canonical interferon response element (5XISRE) (Fig 2B). The transfected cells were treated with interferon beta or infected with AdEGFPuci, and transcriptional activities of the reporter genes were assessed by measurement of luciferase activity at 24 and 72 hours. As expected, treatment with IFN-beta resulted in rapid induction of luciferase activity for the 5XISRE promoter by 24 h, indicative of rapid induction of an antiviral state. The effect persisted since luciferase activity was still elevated at 72 h (compared to mock-treated cultures). In contrast induction of the OAS2 promoter was more modest, ca. 4-fold, compared to the > 40-fold induction for the 5XISRE promoter. In AdEGFPuci infected cells, the pattern of induction was different. The 5XISRE reporter plasmid did not show significant induction compared to the mock-treated cells at 24 h, and there was a very modest ca. 2-fold induction at 72 hr. The OAS2 reporter did not show significant increase in luciferase activity at 24 h, but there was a 5–10 fold increase at 72 hrs. Thus induction of OAS2 expression by adenovirus may involve elements in addition to the canonical ISRE. The luciferase reporter assays of Fig 2B were consistent with the RT-PCR assays of Fig 2A, and they indicate that AdEGFPuci induces a limited antiviral state in 293 cells. The fact that OAS2 expression was higher at 72 h than 24 h could reflect the time required for induction of IFN expression by the viral infection and/or modulation of the IFN response by adenoviral genes.

Since the results of Fig 2 indicated that AdEGFPuci induces a limited antiviral state in 293 cells, we tested if this could be involved in the two types of viral spread observed. Valproic acid (VPA), an inhibitor of histone deacetylases [26], has been shown to inhibit the interferon response in the context of oncolytic herpesviruses [27]. As shown in Fig 2Ci, VPA reduced IFN beta induction of OAS2 RNA in Ad-293 cells at both 24 and 72 h in a dose-dependent manner. Thus it was a suitable inhibitor for these experiments. On the other hand, VPA has been shown to have both negative and positive effects on multiple cellular pathways [28], so it could also have other effects on viral replication. Indeed, the extent of the spreading infections (both robust and limited) were somewhat reduced by VPA (typical examples are shown in Figure A in the Supplementary Materials), and the total numbers of spreading infections were reduced in a dose-dependent fashion (Table 1). Nevertheless VPA increased the proportion of robust vs. limited spreading infections in a dose-dependent manner (Table 1). These results therefore supported the hypothesis that induction of a limited antiviral state by AdEGFPuci was influencing the relative proportion of robust vs. limited virus spread.

**Table 1:** Effect of valproic acid on viral spread^1^.

VPA (mM)	Spreading Infections		
	Limited	Robust	% Robust
0	131	120	48
10	30	54	64
15	16	30	67

^1^Ad-293 cells were infected with AdEGFPuci under conditions of plaque formation, in the presence of different concentrations of valproic acid. At 14 days post-infection the numbers of spreading infections with limited and robust patterns were scored by fluorescent microscopy. The numbers in the table define the number of infection foci that were observed to be limited or robust (e.g. without VPA a total of 251 infection foci were observed, with 131 of them being limited, and 120 robust, equivalent to 48% robust infection foci). Valproic acid treatment significantly increased the percent robust infections (p = 0.0088 for 10mM, and p = 0.03 for 15mM, z-score for comparing population proportions).

Since VPA apparently had other effects besides inhibition of interferon on adenoviral infection, we tested two additional inhibitors of interferon: the mTOR inhibitor rapamycin [29], and a blocking antibody to the interferon alpha receptor 2 (IFNAR2). As shown in Fig 2Cii, both anti—IFNAR2 antibody and rapamycin inhibited IFNβ induction of OAS2 RNA; inhibition by anti—IFNAR2 was rapid (within 24 h), while inhibition by rapamycin was only evident at 72 hr. Experiments analogous to those of Table 1 are shown in Table 2. Treatment with both compounds substantially increased the relative percentage of robust spreading infections, consistent with the conclusion that an interferon response was influencing the relative outcomes of the spreading infections. The effect of the anti-IFNAR2 antibody was particularly noteworthy since it would be expected to be targeting the interferon response quite specifically. In this case the shift from limited to robust spreading outcomes occurred without a change in the total number of spreading infections. Rapamycin treatment reproducibly enhanced the total numbers of spreading infections, but the mechanism of this has not been investigated.

**Table 2:** Effects of anti—ifnar2 and rapamycin on viral spread^1^.

Treatment	Spreading Infections		
	Limited	Robust	% Robust
None	74	68	48
	5	5	50
Anti—IFNAR2	26	105	80
	5	17	77
Rapamycin	93	379	80
	15	48	76

^1^Ad-293 cells were infected with AdEGFPuci under conditions of plaque formation, in the presence of anti-IFNAR2 mAb (1 μg/ml) or rapamycin (5 ng/ml). The infections were carried out with two different concentrations of AdEGFPuci differing by ten-fold. At 14 days post-infection the numbers of spreading infections with limited and robust patterns were scored by fluorescent microscopy. Results for the lower virus inocula are shown below the results for the higher inocula. The numbers in the table define the number of infection foci that were observed to be limited or robust, see legend for Table 1 for more details. Both treatments significantly increased the percent of robust infections (p<<0.001 in both cases, z-score for comparing population proportions). This statistical significance applies to the higher inocula data. No statistics were performed on the lower inocula experiments because the number of resulting infection foci were very limited. Nevertheless, the same trend is observed.

### Mathematical modeling

While experiments suggest that an IFN-induced antiviral state contributes to explaining our observations, we need mathematical models to test whether this is sufficient to explain the simultaneous occurrence of the limited and robust infections under identical conditions. To account for the data, a model would need to be characterized by some form of bistability, with the outcome depending on initial conditions. In a stochastic setting, the dynamics can then randomly enter one or the other domain of attraction, giving rise to different outcomes even when starting from the same initial conditions.

A variety of mathematical models will be built to investigate this. Models of increasing complexity will be examined. First, we will consider ordinary differential equations (ODEs) that assume perfect mixing of viruses and cells. While this does not account for the spatial constraints in our experiments, it is important to start with such models for two reasons: (i) They are analytically more tractable, and the insights we gain from such models can be used to examine the properties of more complex, spatial models. (ii) Such models form the basis of much of the virus dynamics literature [1], and this analysis will indicate whether the dynamics observed in our experiments are particular to the experimental conditions studied here, or whether this is a more broadly applicable phenomenon. Once we have analyzed such models, we will investigate the dynamics in two different, spatially explicit models: a metapopulation model that builds on the ODEs, and a 2-dimensional agent-based model that is most closely connected to our experiments.

### The basic model of virus dynamics

The mathematical models presented here build on a basic virus dynamics model that is well-established in the literature [1] and briefly summarized here. Denoting the number of uninfected target cells by *x* and the number of infected target cells by *y*, the model (hereafter called model (1)) is given by the following pair of ordinary differential equations.

$$\begin{array}{l} \dot{x}=\lambda -dx-\beta xy\\ \dot{y}\;=\;\beta xy-ay \end{array}$$ (1)

Because the free virus population tends to turn over fast relative to the infected cell population, free virus is assumed to be in a quasi-steady state and hence the concentration of free virus is not modeled directly. Uninfected cells are produced with a rate λ, die with a rate d, and become infected by virus with a rate β. Infected cells are assumed to die with a rate a, where *a>d*. This model has two equilibria: the virus extinction equilibrium where x^(0)^ = λ/d, y^(0)^ = 0, and the virus persistence equilibrium where x^(1)^ = a/β, y^(1)^ = λ/a-d/β. In particular the virus persistence equilibrium is stable when the basic reproductive ratio of the virus (R_0_ = (λβ)/(da)) is greater than one. Such a model has been used to describe *in vivo* infection dynamics where target cells are produced with a constant rate. Target cell input with a constant rate, however, does not typically apply to in vitro experiments or to all in vivo tissues, where target cells can divide. Therefore, we will also consider a second version of this model that assumes division of target cells. In this version, instead of the constant input rate λ, we assume density-dependent target cell division, expressed by the term *rx[1-(x+y)/K]*, where r is the replication rate of uninfected cells and K is the carrying capacity of the system. This model will be referred to as model (S1), and more detailed properties are given in the Supplementary Materials. It is important to consider both models to investigate further whether results are limited to assumptions that apply to our experiments, or whether they have more general relevance.

### Modeling anti-viral states with ordinary differential equations

We extend the basic virus dynamics model (1) to include an interferon-induced anti-viral state as follows (the modifications for model (S1) are given in the Supplementary Materials). The uninfected and infected cell populations that are not in an anti-viral state are denoted by x_1_ and y_1_, respectively. Uninfected cells that are in an anti-viral state are denoted by x_0_. We assume that a cell that is in an anti-viral state cannot be productively infected, so infected cells in an anti-viral state are not included in this model. The model is thus given by the following set of ordinary differential equations:

$$\begin{array}{l} {\dot{x}}_{1}=\lambda -dx_{1}+gx_{0}-\beta x_{1}y_{1}-\gamma x_{1}y_{1}\\ {\dot{x}}_{0}=\gamma x_{1}y_{1}-gx_{0}-dx_{0}\\ {\dot{y}}_{1}=\beta x_{1}y_{1}-ay_{1} \end{array}$$ (2)

As in model (1), λ denotes the rate of target cell production and β the rate of infection. Uninfected and infected cells die with rates d and a, respectively. Infected cells can induce an anti-viral state in the uninfected cells with a rate γ, making them resistant to infection. This cell population is assumed to die with a rate d, and can lose its anti-viral state with a rate g. The properties of this model are very similar to those of the basic model (1) without the anti-viral state. The virus-free equilibrium is given by x_1_^(0)^ = λ/d, x_0_^(0)^ = y^(0)^ = 0. Virus persistence is described by the following equilibrium expressions:

$$x_{1}{}^{*}=\frac{a}{\beta };\;x_{0}^{*}=\frac{\gamma (\lambda \beta -da)}{\beta \left(\beta \left(g+d\right)+d\gamma \right)};\;y_{1}^{*}=\frac{g(\lambda \beta -da)+\beta \lambda d-d^{2}a}{a(\beta (g+d)+d\gamma )}.$$

The basic reproductive ratio, R_0_ = (λβ)/(da)), is identical to model (1). As in model (1), there is also no bistability in this model, and when R_0_>1, the virus persistence equilibrium is stable. The same properties hold for the model that assumes density-dependent division of target cells (see model S2 in the Supplementary Materials). These results strongly suggest that for well-mixed systems of cells, an interferon-induced anti-viral state alone cannot explain the occurrence of two alternative outcomes under identical experimental conditions.

### The saturation of anti-viral defenses by multiple infection can account for the experimental observations in ordinary differential equation models

Here, we introduce another layer of complexity into the model: Cells can be infected multiple times by the virus. Multiple infection has been shown to occur in adenovirus infections, and a higher infection multiplicity can result in higher virus output from infected cells [30–34]. If cells in an anti-viral state become infected with multiple viruses, it is possible that this can saturate factors that prevent infection of those cells. Hence, with multiple infection, cells in an antiviral state can still become infected and produce some offspring virus. Modifying our model as follows captures these features:

$$\begin{array}{l} {\dot{x}}_{1}=\lambda -dx_{1}+gx_{0}-\beta x_{1}y_{1}-\gamma x_{1}y_{1}\\ {\dot{x}}_{0}=\gamma x_{1}y_{1}-gx_{0}-\beta x_{0}y_{1}-dx_{0}\\ {\dot{y}}_{1}=\beta (x_{1}+y_{0})y_{1}-ay_{1}\\ {\dot{y}}_{0}=\beta x_{0}y_{1}-ay_{0}-\beta y_{0}y_{1} \end{array}$$ (3)

This model is an extension of model (2) and contains a population of infected cells that are in an anti-viral state, y_0_. For simplicity we assume that two viruses within a cell are sufficient to overcome the anti-viral state. The number of viruses required to overcome the anti-viral state is unknown and can be easily adjusted in the model. Thus, when an uninfected cell in an anti-viral state, x_0_, becomes infected it turns into an infected cell in an anti-viral state that fails to replicate the virus, y_0_. If this cell, however, becomes infected with a second virus, it turns into a productively infected cell. Hence, multiple infection is assumed to completely overcome the anti-viral state and results in a rate of virus production that is identical to cells that are not in an anti-viral state.

**Fig 3:**
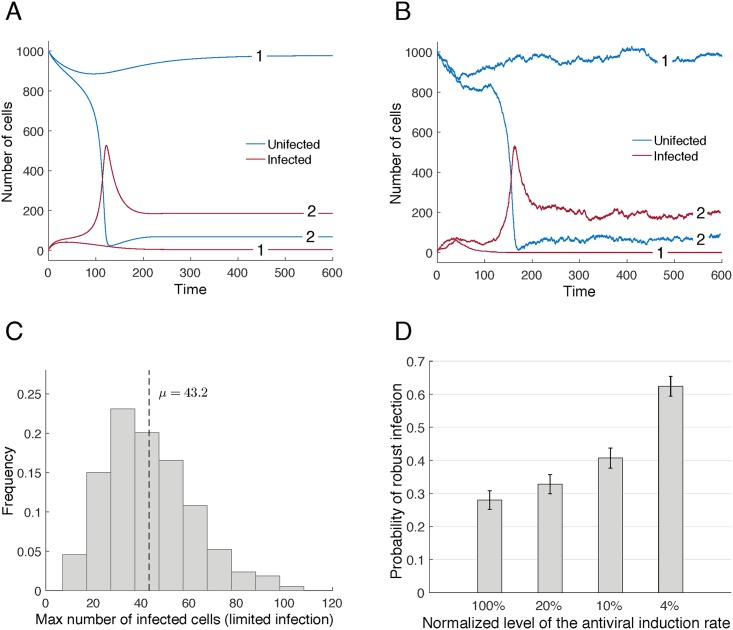
A) Bistability in model (3). The trajectories labeled with “1” depict an example of a weak limited infection. The trajectories labeled with “2” depict a robust viral infection. The only difference between the plots is the initial number of infected cells, four for the limited infection and five for the robust infection. B) Stochastic version of model (3). Panel shows two simulations with identical initial conditions. Stochastic events at early stages of the infection can push the results into in either a weak limited infection that is eventually extinguished (labeled “1”), or in a persistent robust infection that significantly reduces the overall number of cells (labeled “2'”). C) Distribution of the maximum number of infected cells when there is a limited infection. Although the initial number of infected cells is very small (four cells) at their maximum extension limited infections average approximately 43 cells. Results are based on 1000 simulations. D) Probability of the emergence of a robust infection as a function of the normalized level of the antiviral induction rate γ. Results are based on the 1000 simulations for each level of the antiviral induction rate depicted in the panel. Initial conditions: y_1_(0) = 4, and x_1_(0) = 996 (all other cell types equal to zero). Error bars indicate 95% confidence intervals. Parameters: In all panels λ = 10, d = 0.01, β = 0.001, g = 0.1, and a = 0.05; γ = 10 in panels A) and B) and it is equal to 5 in C); in panel D) the 100% level of the antiviral induction rate corresponds to a value of γ = 50.

In the absence of the infection, we again have the trivial steady state, given by x_1_* = λ/d, x_0_* = 0, y_1_* = 0, y_0_* = 0. The basic reproductive ratio of the virus is identical to that in the previous models and thus given by R_0_ = (λβ)/(da). If R_0_<1, then the virus-free equilibrium is stable and the only outcome is virus extinction. If R_0_>1, however, the situation is more complex than in the previous models. The Supplementary Materials show that multiple non-trivial steady states are possible, which, however, are too complicated to define analytically. Hence, this is instead explored with numerical methods. We find that depending on the values of the parameters, there can be multiple non-negative stable steady states. That is, some sets of parameter values result in more than one non-negative stable steady state; while other parameter sets result in just one non-negative stable steady state. Fig 3A depicts an instance where the model is bistable: with the same set of parameters and depending on the initial conditions, the trajectories converge to different equilibria. In this figure, if the initial number of infected cells is four (plots labeled with “1”) the infection first takes off, but eventually stalls and regresses to a number very close to zero. If on the other hand the initial number of infected cells is five (plots labeled with “2”) the trajectories converge to a steady state where the overall number of cells is significantly diminished and infected cells make up a large fraction of the entire cell population. Qualitatively identical properties are found if we assume density-dependent proliferation of uninfected cells, as described in the Supplementary Materials (see model (S3)). A detailed numerical exploration of the parameter space for models (3) and (S3) and the parameter regions that lead to multiply stable outcomes is included in the Supplementary Materials. Interestingly, in the parameter space explorations we found that parameter sets that produce bistable outcomes are more frequent when the antiviral induction rate, γ, is significantly larger than the infection rate, β (see e.g. Figure E(ii) in the Supplementary Materials). IFN molecules are many times smaller than virions [35], and thus should diffuse much faster than them [36]. In the context of our modeling framework this suggests significantly higher values of γ compared to β. Ultimately however, the rates β and γ should be determined experimentally in future studies.

Hence, we conclude that if cells can be induced to be in an anti-viral state by IFN, and if multiple infection of cells can overcome this anti-viral state, then the ODE model can account for the two different experimentally observed outcomes. Depending on the exact initial conditions, either sustained virus growth or limited virus growth can occur for the same parameter sets. If the infection is started from the same initial number of infected cells, then it is possible that stochastic effects push the system in one domain of attraction or the other, thus leading to the alternative infection outcomes. This is explored further in the following section, using a stochastic version of our models.

### Stochastic implementation

Here, we investigate if a stochastic formulation of the previous models can produce the different outcomes under identical initial conditions. The idea behind this hypothesis is that early stochastic events can push the system into either one of the observed infection outcomes. We implement the stochastic formulations in the standard way using Gillespie's algorithm [37].

Fig 3B depicts two stochastic simulations of model (3), which use the same set of parameters and identical initial conditions. In the first simulation (plot labeled with “1”) the infection first takes off, peaks at around 60 infected cells and then starts to regress until it eventually goes extinct. In the second simulation (labeled with “2”) the infection is never extinguished, instead the infection persists at high levels. The same is observed for the model with density-dependent target cell proliferation (see Supplementary Materials Figure B). It is important to note that the infection extinction outcomes are not in general the result of stochastic fluctuations around the initial very low numbers of infected cells. This observation is verified by Fig 3C. This figure presents the distribution of the maximum number of infected cells for simulations where the ultimate outcome was viral extinction. Note that although the initial number of infected cells is very small (four cells) at their maximum extension limited infections average approximately 43 cells. This behavior is even more pronounced in the simulations with the density-dependent target cell proliferation model, where the average maximum number of infected cells for the limited growth was 80 cells (Supplementary Materials, Figure B).

As mentioned above, inhibition of IFN signaling in our experiments resulted in a shift in outcome towards a prevalence of robust infections vs. limited infections. The same qualitative behavior is reproduced by our models. Fig 3D plots the probability of the emergence of a robust infection as a function of the relative strength of the antiviral induction rate, γ. As this figure indicates, the reduction of the strength of the antiviral induction rate, which would occur as a consequence of the down modulation of IFN signaling, results in an increased probability of establishing a robust infection. The same is observed for the model with density-dependent target cell proliferation, as described in the Supplementary Materials (Figure B).

### Dynamics in spatially explicit metapopulation models

To study the dynamics of multiple infection and antiviral response in a spatial setting we consider a meta-population approach, which builds closely on model (3). The metapopulation model consists of a collection of n local patches. Within individual patches, local dynamics occur according to mass-action rules. The patches are coupled to each other by populations migrating between them. Here, we consider a one-dimensional metapopulation model where populations in a given patch can only migrate to the nearest patches. The ODE formulation of the equivalent to model (3) is given as follows:

$$\begin{array}{l} {\dot{x}}_{1,i}=\lambda k-dx_{1,i}+gx_{0,i}-\left(\beta /k\right)x_{1,i}y_{i,1}-\left(\gamma /k\right)x_{1,i}y_{1,i}+\left(m/2\right)\left(x_{1,i-1}-2x_{1,i}+x_{1,i+1}\right)\\ {\dot{x}}_{0,i}=\left(\gamma /k\right)x_{1,i}y_{1,i}-gx_{0,i}-\left(\beta /k\right)x_{0,i}y_{1,i}-dx_{0,i}+\left(m/2\right)\left(x_{0,i-1}-2x_{0,i}+x_{0,i+1}\right)\\ {\dot{y}}_{1,i}=\left(\beta /k\right)\left(x_{1,i}+y_{0,i}\right)y_{1,i}-ay_{1,i}+\left(m/2\right)\left(y_{1,i-1}-2y_{1,i}+y_{1,i+1}\right)\\ {\dot{y}}_{0,i}=\left(\beta /k\right)\left(x_{0,i}-y_{0,i}\right)y_{1,i}-ay_{0,i}+\left(m/2\right)\left(y_{0,i-1}-2y_{0,i}+y_{0,i+1}\right) \end{array}$$ (4)

where k is the carrying capacity of each patch and cells with subscript i refer to cells in the ith patch. We assumed that the patches are arranged in a 1-dimensional linear array, and both target cells and infected cells can migrate to the neighboring patches to the left and to the right of a given patch with migration rate equal to m (the boundary conditions at i = 0 and i = n prevent cell migration outside of the array). The stochastic implementation of the metapopulation model follows directly from the reformulation of model (4) using Gillespie's method.

**Fig 4:**
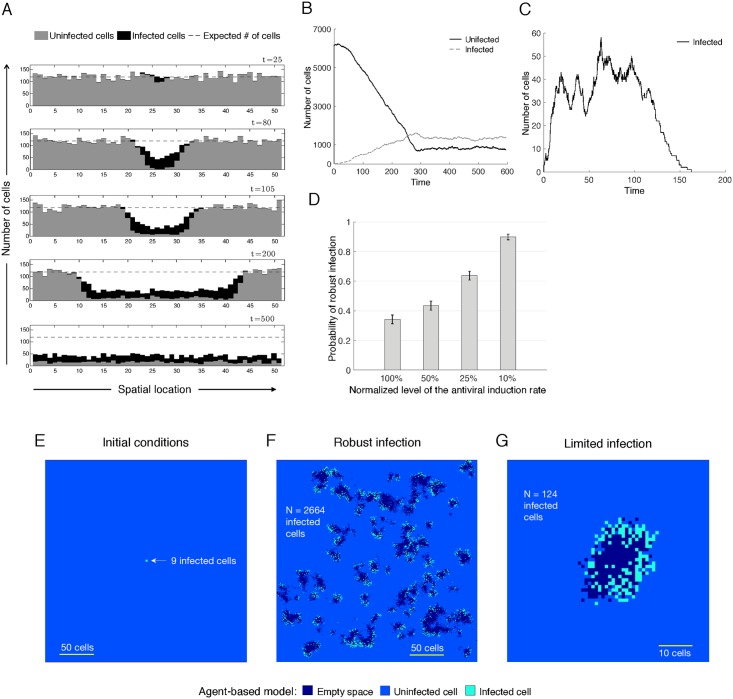
A) Spatial spread of a robust infection in the stochastic metapopulation model based on system (model (4)). There are n = 51 local patches of cells. The expected number of cells in each patch prior to the infection is k = 121 (indicated by the dashed lines). At the start of the simulation all patches contain k uninfected cells and three infected cells are placed in the middle of the spatial array (*i = 26*). B) Time evolution of the infected and uninfected cell populations for the simulation in panel A. The populations refer to the total number of cells in the spatial array. C) Example of a limited infection in the metapopulation model. The infection first takes off, but then stalls and regresses until it is eventually extinguished from the cell population. D) Probability of the emergence of a robust infection as a function of the normalized level of the antiviral induction rate γ. Based on the 1000 simulations for each level of the antiviral induction rate. Error bars indicate 95% confidence intervals. Parameters in panels A-D: λ = 0.0194, d = 0.0194, g = 0.0202, a = 0.0792, β = 1, γ = 10000, and m = 2. Panels (E-G): Robust and limited infections in the two dimensional agent-based model. E) At time t = 0 nine infected cells are placed in the center of a 300 × 300 lattice filled with uninfected cells. Stochastically the simulations result in either limited or robust infections. F) Snapshot of a robust infection, taken at a time point when the system stochastically oscillated around a steady state (see Figure D(i) in Supplementary Materials). Even if there is a ring structure during initial growth, this breaks down over time due to the stochastic dynamics and does not persist in the long-term. G) Snapshot of a limited infection at its maximum extension. Parameters: r = 0.1262, β = 1, γ = 6.238 × 10^4^, g = 18.29, a = 2.403. (See Figure D for the time series of the simulations in the Supplementary Materials).

We find that in the stochastic metapopulation model the two types of infection outcomes (limited vs robust infection) can occur under identical initial conditions and with the same set of parameters (Fig 4B and 4C). Fig 4A illustrates the spatial spread of a robust viral infection. The effect of inhibiting IFN signaling (as done in the experiments) is represented in model (4) through a reduction of the anti-viral induction rate (γ). In agreement with experiments, in the spatial metapopulation model the down modulation of IFN signaling results in an increased proportion of the robust infection outcomes (Fig 4D). The same qualitative results can be obtained in a spatial metapopulation model if we assume density-dependent target cell proliferation (see Supplementary Materials, Figure C).

These explorations show that the induction of an anti-viral state and the ability of multiple infection to overcome the anti-viral state can account for the two infection outcomes observed in the data in a spatial setting, which is closer to our experimental conditions. The advantage of the metapopulation model is that we can build on the insights obtained from the analysis of the ODEs and explore this in a spatial setting. In the next section, we take this work further away from the ODE approach and consider a 2-dimensional stochastic spatial agent-based model of these dynamics.

### Dynamics in a two-dimensional stochastic agent-based model

In the experiments, cells are arranged in a two-dimensional layer, and an agar overlay prevents long-range spread of the virus away from infected cells in the culture medium, creating conditions where virus spread is most likely to occur to neighboring cells. To model these dynamics we create a two-dimensional stochastic agent-based model, where each cell occupies a certain position in a two-dimensional rectangular lattice. There are four types of cells: susceptible uninfected cells (x_1_), uninfected cells in antiviral state (x_0_), infectious cells (y_1_) and infected cells in antiviral state (y_0_). The basic rules are the same as those in the previously explored model. Because this is a model that directly relates to our adenovirus experiments, we will only consider the assumption of density-dependent target cell growth. The rules are described as follows:

- **Infection.** Infectious cells (y_1_) can infect neighbor cells at a rate β. For each kind of susceptible cell, infections result in the following transitions: x_1_→y_1_, x_0_ →y_0_ and y_0_→y_1_.
- **Induction of the antiviral state.** Infectious cells (y_1_) can induce the antiviral state in neighboring uninfected cells at rate γ, (schematically x_1_→ x_0_). The rate of antiviral reversal of uninfected cells (x_0_→x_1_) is g.
- **Cell death.** Infected cells (y_0_ and y_1_) die at a certain rate a. In the duration of the experiments, cell death of uninfected cells was not observed and thus, we do not include cell death of uninfected cells in the simulations.
- **Cell division.** Uninfected cells that are not in the antiviral state (x_1_) reproduce at rate r. Cell division is only possible if there is an unoccupied lattice position in the neighborhood of the dividing cell. Note that adenoviruses lock cells in the S-phase for replication, preventing them from undergoing mitosis [38]. There is also evidence that IFN (which induces antiviral states) prevents cell division [39–42]. This assumption, however, is not likely crucial to our findings, since the results are robust across models that do and do not assume any target cell division.

As we previously mentioned, in the experiments viral spread is restricted to cell neighbors. In the model the neighborhood of a cell is determined by the lattice coordinates that the cell occupies. For the simulations we choose a neighbor radius of two cells in any direction. The model is implemented using the Next Reaction Method [43] (for details see Supplementary Materials).

Consistent with the other models, we observed both limited and robust infection outcomes (depicted in Fig 4E–4G), and that both can occur stochastically under identical parameter combinations and initial conditions. This again required the occurrence of an antiviral state, and the assumption that multiple infection can overcome the antiviral state. The effect of inhibiting IFN signaling is modeled through a reduction of the anti-viral induction rate γ. In agreement with experiments, the down modulation of IFN signaling results in an increased proportion of the robust infection outcomes (Figure D in Supplementary Materials). Statistical results from the simulations and a numerical exploration of the parameter space are found in the Supplementary Materials.

## Discussion and Conclusion

Typically, when virus growth dynamics are investigated, even the early exponential growth phase corresponds to infected cell numbers that are relatively high, where virus levels are readily detectable. This applies to in vitro experiments and even more so to in vivo studies. Our work, however, has shown that complex dynamics can occur when virus concentrations are much lower (starting from a single infected cell), and that these dynamics might play an important role in determining the fate of the infection. In this respect, our experiments identified IFN-induced antiviral states in cells as a crucial mechanism that contributes to the observations. Our mathematical models, however, suggest that this alone cannot explain the experimental outcomes and that additional mechanisms need to be invoked to account for the data. In this respect, we identified the ability of the virus to overcome the anti-viral state by multiple infection as a possible candidate mechanism. Under these assumptions, an initial race between the spread of the virus population and the spread of the antiviral state can stochastically result in two different outcomes under identical conditions. While this is a likely mechanism, and while there is indication that multiple infection of cells might indeed play an important role in adenovirus spread [30–34], we have not been able to explicitly test this notion so far. Hence, one has to be aware that there could potentially be other mechanisms that might also explain the data.

An important question concerns the generality of these notions, i.e. whether the results have relevance beyond the virus-cell system considered in our study. Our mathematical models suggest that such dynamics could be a more general phenomenon. Our experimental system involved a two-dimensional monolayer of cells with agar layover, which is best described by a spatially restricted agent based model. Qualitatively identical results, however, are seen in relatively simple ordinary differential equation models that describe virus dynamics in a setting without any spatial restrictions. Further, the results remain robust in different model formulations. For example, the exact target cell dynamics (production vs. division of target cells) does not appear to change the notions reported here. From an empirical point of view, it remains to be investigated whether multiple infection can saturate an antiviral state in cells, and how general a phenomenon this is. This mechanism can explain the data in the context of our model, and is therefore a model-generated hypothesis. In our experimental system, we demonstrated that AdEGFPuci induced only a limited antiviral state in 293 cells. It is possible that a limited anti-viral state can be overcome more easily by multiple infection than a stronger anti-viral state that may occur in other virus-host cell systems. It would be interesting to test this notion experimentally.

Beyond improving our understanding of the principles of virus dynamics, our results have important practical implications for the field of oncolytic virus therapy of tumors [22,23,44], and add to previous mathematical modeling work that analyzes the dynamics of oncolytic viruses, e.g. [21,45–49]. Some clinically important oncolytic viruses are based on the adenovirus used in our experiments [23]. An important first step in successful oncolytic virus therapy is that the virus establishes a robust infection and efficiently spreads throughout the tumor. The very early spread dynamics might be a crucial phase in this respect, and might pose the first barrier to success. Our study indicates that not only the strength of interferon-induced anti-viral states might be important in this respect, but that a high local infection multiplicity at the earliest stages of the infection process might be equally important for ensuring that this early barrier is crossed and that the infection enters a regime in which robust growth to large viral population sizes is achieved. As a next step, it would be useful to test these notions in the context of different, specific oncolytic viruses that grow on tumor cell lines in vitro. As mentioned above, it is possible that our findings are the result of a limited IFN-induced anti-viral response in 293 cells infected with our virus AdEGFPuci. Hence, it would be interesting to compare the oncolytic virus dynamics in the context of virus-tumor systems in which the strength of an IFN-induced antiviral state varies. For example, some tumors show abnormalities and defects in IFN signaling, and this can in fact be a mechanism for the selective replication of oncolytic viruses in tumor cells [50]. Oncolytic vesicular stomatitis virus (VSV) is an example of this [51]. It would be important to investigate the dynamics studied here in the context of this virus, as well as with other oncolytic viruses that are characterized by different mechanisms of tumor selectivity, and that experience the induction of a stronger IFN-induced antiviral state in tumor cells. Such insights could guide future work that aims to optimize the virus itself as well as the method by which the virus is delivered to the cancer.

## Materials and Methods

### Cells and viruses

Ad-293 cells, derivatives of HEK293 cells that express adenovirus EIA and EIB proteins, were purchased from Agilent Technologies (La Jolla, CA) as described previously [20]. AdEGFPuci, a recombinant adenovirus expressing enhanced jellyfish green fluorescent protein in place of EIA and EIB was also described previously [20]. Stocks of 10^9^−10^11^ pfu/ml were used.

### Infections

Infections of AdEGFPuci onto Ad-293 cells on gridded culture dishes were performed at different multiplicities under conditions of plaque formation as described previously [20]. Infections were monitored by fluorescence microscopy for GFP; areas of infection were counted and scored as limited or robust. The same dishes were scored at different days post-infection.

### RT-PCRs

Total RNA from infected or uninfected Ad-293 cells was extracted using TRIzol (Life Technologies), and 2 μg of RNA was digested with DNAse I and converted to cDNA using the qScript cDNA synthesis kit (Quantas) according to the manufacturer’s instructions. For detecting induction of cellular interferon response genes, PCR primer sets for different cellular genes from the Interferon Response Detection Kit (Systems Biosciences) were employed and PCR amplifications for different cycles were carried out according the manufacturer’s instructions. PCR products were visualized by agarose gel electrophoresis and ethidium bromide staining. For quantitative RT-PCR of OAS2 RNA, the following human OAS2 primers were used: 5’AGCTCCTCCTTTTTCCTTCCAGTC3’ (forward) and 5’TGGCTGGCTGCTGGCATAGAG3’ (reverse).

For standardization, the following human GAPDH primers were used: 5’CAACTACATGGTCTACATGTTC3’ (forward) and 5’ctcgctcctggaagatg3’ (reverse).

Quantitative RT-PCRs were performed using Power SYBR green PCR master mix with the 7900HT Fast real-time PCR system (Applied Biosystems) according to the manufacturer’s instructions. All qRT-PCRs were run in triplicate. The RNA expression levels were determined by the relative comparative threshold cycle (C_r_) method.

### Luciferase assays

To measure transcriptional activities, the following firefly luciferase reporter plasmids were used. Path Detect ISRE-luc (Agilent technologies) is a luciferase reporter driven by 5 tandem copies of an Interferon response element (ISRE), and is referred to here as 5XISRE-luc. The matched plasmid lacking promoter or enhancers, pCIS-CK (Agilent), was used as a negative control. A luciferase reporter driven by the upstream control elements of the 2’-5’- oligo (A) synthetase 2 (OAS2) gene was generated by first PCR amplifying OAS2 sequences (–880 to +197) from 293 cell DNA using synthetic primers containing sites for Hind III and Bgl II at either end. The PCR fragment was digested with Hind III and Bgl II, and cloned into the pLuc-MCS plasmid (Agilent) between Hind III and Bgl II sites in the multiple cloning site of the plasmid. This plasmid was designated OAS2-luc.

Ad-293 cells were transfected with the different luciferase reporter plasmids as described previously [52], with or without interferon treatment or infection with AdEGFPuci. Cell lysates were prepared and analyzed for luciferase activity as described previously [52], using the dual luciferase assay kit (Promega). Luciferase activities were read in a Sirius-L luminometer (Berthold Detection Systems); transfections were performed in triplicate, and each assay was performed at least three times.

### Reagents

Human interferon beta was purchased from Pepro Tech, Interferon alpha receptor 2 antibody from Life Techologies, valproic acid from Sigma-Aldrich, and rapamycin from Fisher Scientific.
