# Correction: Complex Dynamics of Virus Spread from Low Infection Multiplicities: Implications for the Spread of Oncolytic Viruses

**DOI:** 10.1371/journal.pcbi.1005548

**Published:** 2017-05-19

**Authors:** The PLOS Computational Biology Staff

The Supporting Information section is omitted. The publisher apologizes for the error. Please view the Supporting Information section here:

## Supporting information

S1 Text Supplementary materials. This file includes supplementary data.
(PDF)
