# Supplementary material for: Correction: Complex Dynamics of Virus Spread from Low Infection Multiplicities: Implications for the Spread of Oncolytic Viruses
Source: PLoS Comput Biol. 2017 May 19;13(5):e1005548. doi: 10.1371/journal.pcbi.1005548 (PMC5438102; doi:10.1371/journal.pcbi.1005548)
Supplement: S1 Text — This file includes supplementary data. (PDF) [file pcbi.1005548.s001.pdf]

Supplementary Materials for the article: Complex dynamics of virus spread from low infection multiplicities

Ignacio A. Rodriguez-Brenes[1,2], Andrew Hofacre[3], Hung Fan[3], and Dominik Wodarz[2,1]

[1]Department of Mathematics, [2]Department of Ecology and Evolutionary Biology, [3]Department of Molecular Biology and Biochemistry, University of California, Irvine, CA 92697, USA.

Part A. Supplemental experimental figure

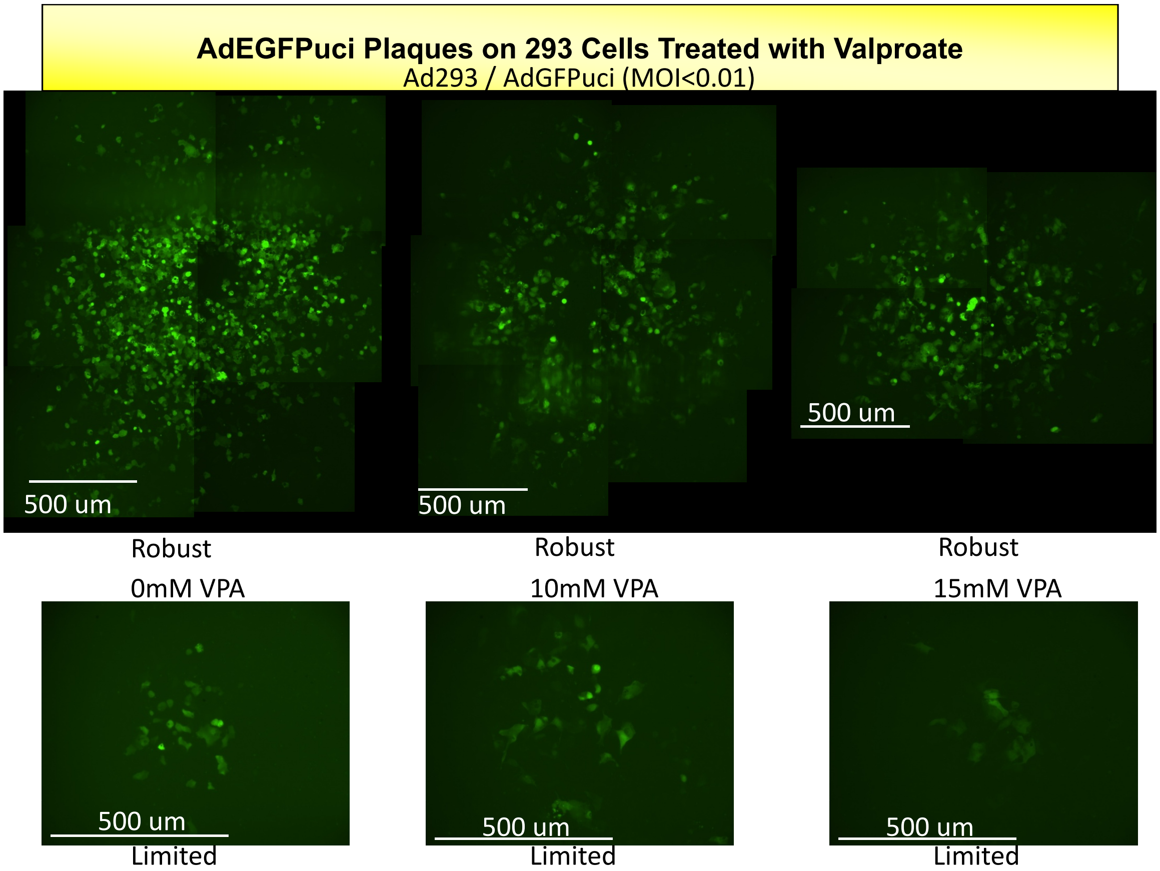

Figure A: Effect of VPA on Infection Spread. Ad-293 cells were infected with AdEGFPuci in the presence of different concentrations of valproic acid. The sizes of typical robust and limited spread areas of infection are shown at 12 days post-infection. The bars indicate 500 μm. VPA reduced the spread of infection in a dose-dependent manner, for both limited and robust infection outcomes. Three robust and three limited infection outcomes are shown as representative examples of the experiments (> 100). See also Table 2.

Part B. Mathematical details

Background models

We will first consider two widely used models of viral infection (Eqs. [1] and [2]) [Perelson and Ribeiro, 2013, Nowak and May, 2000]. These two models distinguish between two types of cell populations: uninfected cells, x_1, and infected cells, y_1. The infection rate is β, the death rate of uninfected cells is d and the death rate of infected cells is a (where $a \geq d$).

Model (1): The first model (Eq. [1]) assumes that uninfected cells are produced with a constant rate λ. This assumption is used to describe *in vivo* infection dynamics where target cells (x_1) are produced by other types of cells not directly modeled in the equations, or when the number of uninfected cells is large and the quantity x_1 represents a smaller subpopulation of these cells.

$$\begin{cases} \dot{x}_1 &= \lambda - dx_1 - \beta x_1 y_1 \\ \dot{y}_1 &= \beta x_1 y_1 - a y_1 \end{cases} \qquad [1]$$

Model (S1): The second model (Eq. [2]) describes a closed system that assumes density-dependent target cell division. This type of model is appropriate to describe the dynamics of *in vitro* colonies of cells. Note that adenoviruses lock cells into the S-phase of the cell cycle preventing them from undergoing mitosis, hence in the model only uninfected cells are able to divide. If r is the division rate and K is the carrying capacity of the system. Model S1 is described by the following equations:

$$\begin{cases} \dot{x}_1 &= r x_1 \left(1 - \frac{x_1 + y_1}{K}\right) - dx_1 - \beta x_1 y_1 \\ \dot{y}_1 &= \beta x_1 y_1 - a y_1 \end{cases} \qquad [2]$$

Stability and equilibria of the background models

The experimental data discussed in the main manuscript describes a biological system where there is more than one stable outcome: weak self-limiting infection vs. robust and persistent viral spread. It is then relevant to analyze the stability of the classical models of viral spread (1) and (S1). We find that both models have at most one non-negative stable steady state.

The steady states of system [1] are $P_1 = (\lambda/d, 0)$ and $P_2 = (a/\beta, (-ad + \beta\lambda)/(a\beta))$. Note that P_2 is non-negative if and only if $\Delta_1 = -ad + \beta\lambda \geq 0$. However, if $\Delta_1 = 0$, then $P_1 = P_2$. Hence, if P_1 and P_2 are different and non-negative we must have $\Delta_1 > 0$. On the other hand the eigenvalues of the Jacobian of [1] evaluated at P_1 are $-d$ and $-a + \beta\lambda/d$. The second eigenvalue is positive when $\Delta_1 > 0$. Hence, P_1 and P_2 cannot be both different, non-negative and stable.

Similarly, the steady states of system [2] are $Q_1 = (a/\beta, (-ar + \beta K(-d + r))/(\beta^2 K + \beta r))$, $Q_2 = (0, 0)$ and $Q_3 = (K - dK/r, 0)$. First, note that the steady state number of cells when there is no infection is $K(1 - d/r)$ and it follows that for this model to be biologically meaningful we must have $d < r$; however, one of the eigenvalues of Q_2 is $r - d$ and thus the trivial steady state, Q_2, is never stable. Next we turn our attention to Q_1 and Q_3. The point Q_1 non-negative if and only if $\Delta_2 = -ar + \beta K(-d + r) \geq 0$. However, if $\Delta_2 = 0$, then $Q_1 = Q_3$. Hence, if Q_1 and Q_3 are

different and non-negative we must have $\Delta_2 > 0$. Now, one of the eigenvalues of Q_3 is Δ_2/r and thus it is impossible to have $Q_1 \neq Q_3$, both points non-negative, and Q_3 stable. It follows that system (2) has at most one positive stable steady state[1].

Modeling the antiviral state

To incorporate the antiviral state in the models we begin by assuming the simplest scenario, i.e., cells in antiviral cannot be infected. Let us call cells in antiviral state x_0, the rate of antiviral induction γ, and the rate of of reversal from the antiviral state g. Then by modifying models (1) and (S1), we find the models (2) and (S2).

Model (2):

$$\begin{cases} \dot{x}_1 &= \lambda - dx_1 + gx_0 - \beta x_1 y_1 - \gamma x_1 y_1 \\ \dot{x}_0 &= \gamma x_1 y_1 - gx_0 - dx_0 \\ \dot{y}_1 &= \beta x_1 y_1 - a y_1 \end{cases} \qquad [3]$$

Model (S2):

$$\begin{cases} \dot{x}_1 &= rx_1\left(1 - \frac{x_1+x_0+y_1}{K}\right) - dx_1 + gx_0 - \beta x_1 y_1 - \gamma x_1 y_1 \\ \dot{x}_0 &= \gamma x_1 y_1 - gx_0 - dx_0 \\ \dot{y}_1 &= \beta x_1 y_1 - a y_1 \end{cases} \qquad [4]$$

Once again we find that models (2) and (S2) have each at most one non-negative stable steady state. The analysis proceeds in a similar way to the discussion found in the previous section. Model (S2) has three steady states: $Q_1 = (0,0,0)$, $Q_2 = (K(1-d/r),0,0)$ and $Q_3 = (\tilde{x}_1, \tilde{x}_0, \tilde{y}_1)$, where

$$\tilde{x}_1 = a/\beta \qquad [5]$$

$$\tilde{x}_0 = \frac{a\gamma r(-a + \beta K(1-d/r))}{\beta\left(a\gamma r + \beta^2 K(d+g) + \beta(d(\gamma K + r) + gr)\right)} \qquad [6]$$

$$\tilde{y}_1 = \frac{r(d+g)(-a + \beta K(1-d/r))}{a\gamma r + \beta^2 K(d+g) + \beta(d(\gamma K + r) + gr)} \qquad [7]$$

Since systems [2] and [4] are identical when there are no infected cells, it follows again that we must have $r > d$ and thus, since one of the eigenvalues of Q_1 is $r - d$, this point cannot be stable. From the expression for $\tilde{x}_0$ in [6] and the definition of Q_2, it follows that Q_3 is non-negative and different from Q_2 if and only if $-a + \beta K(1-d/r) > 0$. However, $-a + \beta K(1-d/r)$ is also an eigenvalue of Q_2 and hence, Q_2 and Q_3 cannot both different, non-negative and stable. A similar argument proves that model (2) has at most one non-negative stable steady state.

[1]In fact, it is easy to prove that each systems has exactly one non-negative stable steady state when Δ_1 and Δ_2 are different than zero.

Saturation of anti-viral defenses by multiple infection

The previous analysis suggests that multiple stability in the model is difficult to obtain if antiviral cells are completely immune to infection. Another possibility is that cells in the antiviral state can still become infected, but the antiviral state inhibits virus production. In this case multiple infection by more than one virus particle can increase the amount of virus produced and eventually saturate the intracellular defenses, causing multiply infected cells in the antiviral state to become infectious. The number of viruses particles that need to enter the cell before the antiviral defenses are overridden is not known. Hence, the first and simplest assumption to investigate is that infection by two or more virus particles overrides the antiviral state. If we adapt models (2) and (S2) to incorporate these set of assumptions we arrive at equations [8] and [9], where y_0 is the number of cells in antiviral state infected by just one virus particle. Note that y_0 cells are not infectious, but once they are infected a second time they turn infectious and behave like y_1 cells.

Model (3):

$$\begin{cases} \dot{x}_1 &= \lambda - dx_1 + gx_0 - \beta x_1 y_1 - \gamma x_1 y_1 \\ \dot{x}_0 &= \gamma x_1 y_1 - gx_0 - \beta x_0 y_1 - dx_0 \\ \dot{y}_1 &= \beta(x_1 + y_0)y_1 - ay_1 \\ \dot{y}_0 &= \beta x_0 y_1 - ay_0 - \beta y_0 y_1 \end{cases} \quad [8]$$

Model (S3):

$$\begin{cases} \dot{x}_1 &= rx_1\left(1 - \frac{x_1+x_0+y_1+y_0}{K}\right) - dx_1 + gx_0 - \beta x_1 y_1 - \gamma x_1 y_1 \\ \dot{x}_0 &= \gamma x_1 y_1 - gx_0 - \beta x_0 y_1 - dx_0 \\ \dot{y}_1 &= \beta(x_1 + y_0)y_1 - ay_1 \\ \dot{y}_0 &= \beta x_0 y_1 - ay_0 - \beta y_0 y_1 \end{cases} \quad [9]$$

We first focus on model (3). The system's trivial steady state is: $P_1 = (\lambda/d, 0, 0, 0)$. This point is asymptotically stable if and only if the basic reproductive ratio of the virus $R_0 = (\lambda\beta)/(da)$ is less than one. Let us know look for the other steady states. First, if for a steady state P we have $y_1 = 0$, it is straightforward to see that $P = P_1$. Hence, we can assume that $y_1 \neq 0$ and setting $\dot{y}_1 = 0$ in Eq. [8] we have:

$$y_0 = \frac{a}{\beta} - x_1 \quad [10]$$

Using the previous equation for y_0 and setting $\dot{y}_0 = 0$ we have:

$$y_1 = \frac{ay_0}{\beta(x_0 - y_0)} = \frac{a(a - \beta x_1)}{\beta(-a + \beta(x_0 + x_1))} \quad [11]$$

Substituting these expressions for y_0 and y_1 into the equation for $\dot{x}_1 = 0$, we find:

$$0 = \lambda - dx_1 + gx_0 - \frac{a(\beta + \gamma)(a - \beta x_1)x_1}{\beta(-a + \beta(x_0 + x_1))} \quad [12]$$

$$\Rightarrow\ 0 = (\lambda - dx_1 + gx_0)(\beta(-a + \beta(x_0 + x_1))) - a(\beta + \gamma)(a - \beta x_1)x_1 \quad [13]$$

Note that the previous equations are not valid when $x_0 - y_0 = -a/\beta + (x_0 + x_1) = 0$. However, if this equality holds, it is straightforward to verify that there is a steady state only if $y_1 = 0$, in

which case we simply recover the trivial steady state P_1.

Expression (13) is a quadratic equation on x_0 and x_1, which can be rewritten as:

$$A_1x_1^2 + B_1x_1x_0 + C_1x_0^2 + D_1x_1 + E_1x_0 + F_1 = 0 \qquad [14]$$

where, $A_1 = \beta^2(a-d)+\beta a\gamma$, $B_1 = \beta^2(g-d)$, $C_1 = \beta^2 g$, $D_1 = \beta^2\lambda+\beta a(d-a)-a^2\gamma$, $E_1 = \beta^2\lambda-\beta ag$ and $F_1 = -\beta a\lambda$. Similarly substituting (10) and (11) into the equation for $\dot{x}_1 = 0$ we find the condition:

$$A_0x_1^2 + B_0x_1x_0 + C_0x_0^2 + D_0x_1 + E_0x_0 + F_0 = 0 \qquad [15]$$

where, $A_0 = \beta a\gamma$, $B_0 = \beta^2(-a+d+g)$, $C_0 = \beta^2(d+g)$, $D_0 = -a^2\gamma$, $E_0 = -\beta(-a+d+g)a$ and $F_0 = 0$. Hence, the non-trivial steady states lies in the intersection of the two conic sections (14) and (15). Two conic sections intersect in 0,1,2 or 4 points, which gives the possible number of non-trivial steady states of the model. An explicit solution for the intersection of two conic sections can be written down. However, the formulas are too unwieldy to be of practical use. Instead we use numerical methods to find and classify the non-trivial steady states according to their stability (see the Numerical Method section in this Supplementary Materials).

We find that depending on the values of the parameters, model (3) can have multiple non-negative stable steady states. That is, some sets of parameter values result in system [8] having more than one non-negative stable steady state; while other parameter sets result in system [8] having just one non-negative stable steady state. See Figure 4 in the main manuscript for an instance where the model is bistable.

Next we turn our attention to model (S3). In this case the trivial steady states are $P_0 = (0,0,0,0)$ and $P_1 = (K(1-d/r),0,0,0)$, where again we have $d < r$. Since one of the eigenvalues of P_0 is $r-d$ it follows that this point is not stable. It is also easy to see that P_1 is asymptotically stable if and only if $K(1-d/r) < a/\beta$.

To analyze the non-trivial steady states, we first note that systems [8] and [9] differ only in their equations for $\dot{x}_1$. It then follows that equations [10], [11] and [13] hold for both models. Thus, the non-trivial steady states of model (S3) lie in the intersection of the conic sections defined by [15] and the equation:

$$0 = H_1\, x_0x_1^2 + H_2\, x_1^2 + H_3\, x_0^2x_1 + H_4\, x_0x_1 + H_5\, x_1 + H_6\, x_0^2 + H_7\, x_0 \qquad [16]$$

where, $H_1 = -\beta^2 r$, $H_2 = \beta K(a(\beta+\gamma)+\beta(-d+r))$, $H_3 = -\beta^2 r$, $H_4 = \beta^2 K(-d+g+r)$, $H_5 = -aK(a(\beta+\gamma)+\beta(-d+r))$, $H_6 = \beta^2 gK$ and $H_7 = -a\beta gK$. Once again it is possible to write explicit solutions for the steady states, but the expressions are to unwieldy to be useful. Instead we use numerical methods to analyze the stability and non-negativity of the non-trivial steady states (see methods section).

We find that depending on the values of the parameters, model (S3) can have multiple non-negative stable steady states, or just one such steady state. Figure B(i) depicts an instance where the system is bistable. With the same set of parameters, but with different initial conditions, the

trajectories converge to two different equilibria. In this figure, when the initial number of infected cells is four (plot labeled with "1") the infection first takes off, but eventually stalls and regresses to a very low level; when the initial number of infected cells is five (plots labeled with "2"), the infection is significantly more robust and it persists affecting a large fraction of the cell population.

Stochastic formulation

In the experiments the different infection outcomes (limited vs robust infections) occur under identical conditions. Hence, we investigate if a stochastic formulation of the previous models can produce the different outcomes under identical initial conditions. The idea being that early stochastic events can push the system into either one of the domains of attraction of each of the two infection outcomes. We implement the stochastic formulations in the standard way using Gillespie's algorithm [Gillespie, 1977].

Figure B(ii) depicts two stochastic simulations of model (S3), both of which use the same set of parameters and identical initial conditions. In the first simulation (plots labeled with "1") the infection first takes and at remains at a low level for a long time until it eventually goes extinct. In the second simulation (labeled with "2") the infection is never extinguished, instead the total number of cells significantly diminishes and infection persists at high levels. It is important to note that the infection extinction outcomes are not in general the result of stochastic fluctuations around the initial very low numbers of infected cells. This observation is verified by Figure B(iii). This figure present the distribution of the maximum number of infected cells for simulations where the ultimate outcome was viral extinction. Note that although the initial number of infected cells is very small (four cells) at their maximum extension limited infections average approximately 80 cells. Analogous results for the stochastic version of model (3) are found in the main manuscript.

As explained in the main manuscript, the inhibition of IFN signaling in our experiments resulted in a shift in outcome towards a prevalence of robust infections vs. limited infections. The same qualitative behavior is reproduced by the stochastic version of model (S3). Figure B(iv) plots the probability of the emergence of a robust infection as a function of the relative strength of the antiviral induction rate, γ. As this figure indicates, the reduction of the strength of the antiviral induction rate, which would occur as a consequence of the down modulation of IFN signaling, results in an increased probability of establishing a robust infection. The same qualitative behavior occurs for model (3) (discussed in the main manuscript).

Metapopulation models

To study the dynamics of the multiple infection and antiviral response in a spatial setting we consider a meta population approach. The model consists of a collection of n local patches. Within individual patches, local dynamics occur according to mass-action rules. The patches are coupled to each other by populations migrating between them. Here, we consider one-dimensional meta population models where populations in a given patch can only migrate to the nearest patches. In the equations k is the carrying capacity of each patch and cell populations with subscript i refer to cells in the ith patch. We assumed that the patches are arranged in a 1-dimensional linear array, and both target cells and infected cells can migrate to the neighboring patches to the left

and to the right of a given patch with migration rate equal to m. The stochastic implementation of the metapopulation models follows directly from the reformulation of models (4) and (S4) using Gillespie's method.

The metapopulation model (4), which assumes a constant production of uninfected cells is described in the main text. Here we present the metapopulation model, which assumes density-dependent target cell division, which we call model (S4). First, let us define the operator $f(z, i) = z_{i-1} - 2z_i + z_{i+1}$ when $0 < i < n$, $f(z, i) = z_{i+1} - z_i$ when $i = 0$ and $f(z, i) = z_{i-1} - z_i$ when $i = n$, then the metapopulation model (S4) is given by Eq. [17].

Model (S4):

$$\left\{\begin{array}{lcl} \dot{x}_{1,i} & = & r x_{0,i}(1 - \frac{x_{1,i}+x_{0,i}+y_{1,i}+y_{0,i}}{k}) - d x_{1,i} + g x_{0,i} - (\beta/k + \gamma/k) x_{1,i}\, y_{i,1} + (m/2) f(x_1, i) \\ \dot{x}_{0,i} & = & (\gamma/k) x_{1,i}\, y_{1,i} - g x_{0,i} - (\beta/k) x_{0,i}\, y_{1,i} - d x_{0,i} + (m/2) f(x_0, i) \\ \dot{y}_{1,i} & = & (\beta/k)(x_{1,i} + y_{0,i})\, y_{1,i} - a y_{1,i} + (m/2) f(y_1, i) \\ \dot{y}_{0,i} & = & (\beta/k)(x_{0,i} - y_{0,i})\, y_{1,i} - a y_{0,i} + (m/2) f(y_0, i) \end{array}\right. \tag{17}$$

Figure C shows simulations from the stochastic implementation of system [17]. Panel (i): An example of the spatial spread of a robust infection for model (S4). Panel (ii): time series for a limited infection. Panel (iii) time series for the number of infected cells in a limited infection.

Two-dimensional stochastic agent-based model

The rationale and algorithmic description of the agent-based model is discussed in the main manuscript. In particular, panels E-G in Figure 4 (main manuscript) depict snapshots of the initial conditions and of robust and limited infections for the 2D agent-based model. Figures D(i) (robust) and D(ii) (limited) plot the time series for Figure 4(E-G). Figure D(iii) depicts the distribution of the maximum number of infected cells for simulations where the ultimate outcome was viral extinction (limited infections). The distinction between limited and robust infection outcomes is more difficult to make in the agent-based model because we cannot use ODEs as a guide to inform us about the stability properties of the the system. Hence, we chose the the threshold of 1000 infected cells to distinguish between robust and limited infections. If the cell population reached 1000 infected cells it was scored as robust, if it did not reach 1000 infected cells before going extinct it was scored as limited. In the experiments limited infections peaked at less than 1000 cells before they started to regress. With these criteria we found that out of 10,000 simulations 4094 resulted in limited infections. The effect of inhibiting IFN signaling was modeled through a reduction of the anti-viral induction rate (γ). In agreement with experiments the down modulation of IFN signaling results in an increased proportion of the robust infection outcomes (Figure D(iv)).

Implementation of the agent-based model

Next, we provide details on the implementation of the two-dimensional stochastic model. First, in the simulation we choose a lattice distance of two cells to define local neighborhoods (i.e., if a cell has a lattice coordinates (i, j) its neighbors have coordinates (k, l), where $i - 2 \leq k \leq i + 2$ and $j - 2 \leq l \leq j + 2$). Let us refer to all cells that can be infected as susceptible cells (types x_0, x_1, y_0),

and to cells that can transmit the virus as infectious cells (type y_1). For each lattice location (i, j) we define four quantities: R_{ij}, I_{ij}, D_{ij} and A_{ij}. The algorithm proceeds as follows: I) If there is a type x_0 cell at location (i, j), make R_{ij} equal to the number of unoccupied lattice point in its neighborhood, otherwise make R_{ij} equal to zero. II) If there is an infectious cell at location (i, j), make I_{ij} equal to the number of susceptible cell in its neighborhood, otherwise make I_{ij} equal to zero. III) If there is an infected cell at location (i, j), make $D_{ij} = 1$, otherwise make $D_{ij} = 0$. IV) If there is an infectious cell at location (i, j), make A_{ij} equal to the number of type x_0 cells in the neighborhood, otherwise make A_{ij} equal to zero. Now, if we define $a = rR_{ij} + \beta I_{ij} + dD_{ij} + \gamma A_{ij}$, then the probability that at location (i, j) the next reaction will be cell death is dD_{ij}/a, and the probability that it will be reproduction is rR_{ij}/a. Similarly, the probability that the next event is infection by a cell in location (i, j) is $\beta I_{ij}/a$, and the probability that it is the conferral of the antiviral state is $\gamma A_{i,j}/a$. In case of reproduction, the placement of one of the two daughters cells is chosen randomly from the unoccupied locations in the neighborhood of the dividing cell. Finally, if a cell at location (i, j) either infects or confers the antiviral state to another cell, the target cell is chosen randomly amongst the suitable cells in the neighborhood. To iteratively select the time and identity of the next reaction, we build and indexed priority queue and a reaction dependency graph as outlined in the so called "Next reaction method" [Gibson and Bruck, 2000]. This method has a $O(\log(m))$ time complexity, where m is the number of lattice sites. Thus the method significantly improves the computational performance of the algorithm used in our previous study, which had a $O(m)$ time complexity. The algorithm was coded in C using subroutines from GNU Scientific Library.

Numerical Method

As we previously mentioned, we can write closed analytical expressions for the steady states of models (3) and (S3), however they are too unwieldy to be of practical use. Hence, we need a robust and efficient numerical method to establish whether a parameter set yields a system with multiple stable equilibria or not. This is particularly important to be able to conduct thorough parameter explorations as the ones conducted in the next sections.

We now describe a method to find and score the equilibria of models (3) and (S3). We will explain the method for system [8]. We have shown the steady states must lie in the intersection of the two conic sections described by equations [14] and [15]. Note that for a fixed x_0 equation [15] is a quadratic equation on x_1, which will have real solutions if and only if its discriminant $\Delta = (B_0x_0 + D_0)^2 - 4A_0(Cx_0^2 + E_0x_0 + F_0) \geq 0$. It is easy to see that for non-negative steady states we must have $0 \leq x_0 \leq \lambda/d$. Since Δ is a quadratic function of x_0, on the interval $[0, \lambda/d]$, the condition $\Delta \geq 0$ defines a set of points D, which is equal to either: i) a closed interval, ii) the union of two disjoint closed intervals, iii) a single point, or iv) the empty set. On the set D equation [15] defines two branches of x_1 as a real-valued functions of x_0, corresponding to the two quadratic solutions for x_1 of [15]. On each branch of x_1 we can then define a function $g : D \to \mathbb{R}$ where $g(x_0, x_1(x_0))$ is equal to the righthand side of equation [14]. If they exist, the non-trivial steady states must be zeros of the functions g. Since, for the non-degenerate cases D is either a closed interval or the union of closed intervals and, the functions g are continuous and smooth in the interior of D, it is straightforward to adapt a conventional one-dimensional search algorithm (e.g., bisection, interpolation, newton's method, etc) to find all the roots. Finally, to evaluate if

the roots are non-negative exponentially stable steady states of [8], we evaluate x_1, y_1 and y_0 for non-negativity and compute the eigenvalues numerically.

Non-negativity of solutions

To establish that the models are well posed from a biological perspective it is important to demonstrate the non-negativity of the solutions. We do so here for models (3) and (S3). The non-negativity of all other ODE based models follows directly from the same type of arguments presented here.

PROPOSITION 1. *"The solution $z(t) = (x_1(t), x_0(t), y_1(t), y_0(t))$ of [8] with initial conditions $x_1(0) > 0$, $x_0(0) = 0$, $y_1(0) > 0$ and $y_0(0) = 0$ is non-negative, i.e.: $z(t) \geq 0$ $\forall t \geq 0$."*

Proof: We will first show that there is an $\varepsilon > 0$ such that $z(t) > 0$ for $t \in (0, \varepsilon]$. First, since $x_1(0)$ and $y_1(0)$ are both positive, and x_1 and y_1 are continuous, there are positive numbers ε_{x_1} and ε_{x_1} such that $x_1(t) > 0$ for $t \in (0, \varepsilon_{x_1}]$ and $y_1(t) > 0$ for $t \in (0, \varepsilon_{y_1}]$. Second, since $x_0(0) = 0$ and $\dot{x}_0(0) > 0$, there is a $\varepsilon_{x_0} > 0$ such that $x_0(t) > 0$ for $t \in (0, \varepsilon_{x_0}]$. Third, since $y_0(0) = 0$, $\dot{y}_0(0) = 0$ and $\ddot{y}_0(0) > 0$, there is $\varepsilon_{y_0} > 0$, such that $y_0(t) > 0$ for $t \in (0, \varepsilon_{y_0}]$. Hence, if we make $\varepsilon = \min\{\varepsilon_{x_1}, \varepsilon_{x_0}, \varepsilon_{y_1}, \varepsilon_{y_0}\}$, $z(t) > 0$ for $t \in (0, \varepsilon]$.

Let $S = \{t \in \mathbb{R}^+ : z(s) > 0 \text{ for } s \in (0, t]\}$. Suppose the proposition is not true (i.e., $z(\hat{t}\,) \ngeq 0$ for some $\hat{t} > 0$), then the set S is non-empty and bounded and thus has a least upper bound $\bar{t} > 0$. Since $z(t)$ is continuous we must have $z(\bar{t}) \geq 0$ and at least one of the entries of $z(\bar{t})$ must be equal to zero.

- Case 1 ($x_1(\bar{t}) = 0$). If $x_1(\bar{t}) = 0$ then $\dot{x}_1(\bar{t}) = \lambda + g x_0(\bar{t}) > 0$, which means $x_1(t)$ is strictly increasing in a neighborhood of $\bar{t}$, but then $\exists\, t^*$ such that $0 < t^* < \bar{t}$ and $x_1(t^*) < x_1(\bar{t}) = 0$, violating the definition of $\bar{t}$ as the supremum of S. Hence, we cannot have $x(\bar{t}) > 0$.
- Case 2 ($y_1(\bar{t}) = 0$). Since $y_1 = 0$ is a steady state of $y_1(t)$, if $y_1(\bar{t}) = 0 \Rightarrow y_1(t) = 0$ $\forall t \geq \bar{t}$. It follows that for $t \geq \bar{t}$: $y_0(t) = y_0(\bar{t}) e^{-a(t-\bar{t})} \geq 0$, $x_0(t) = x_0(\bar{t}) e^{-(g+d)(t-\bar{t})} \geq 0$, and $x_1(t) = x_0(\bar{t}) e^{-d(t-\bar{t})} \left(1 - e^{-g(t-\bar{t})}\right) + x_1(\bar{t}) e^{-d(t-\bar{t})} + (\lambda/d) \left(1 - e^{-d(t-\bar{t})}\right) \geq 0$. Hence, when $y_1(\bar{t}) = 0$, we have $z(t) \geq 0$ for $t \geq 0$.
- Case 3 ($x_0(\bar{t}) = 0$). If $y_1(\bar{t}) = 0$, then $z(t) \geq 0$ $\forall t \geq 0$ and there is nothing to prove. If $y_1(\bar{t}) > 0$, since $x_1(\bar{t}) > 0$, we have $\dot{x}_0(\bar{t}) > 0 \Rightarrow \exists\, t^*$ such that $0 < t^* < \bar{t}$ and $x_0(t^*) < x_0(\bar{t}) = 0$, violating the definition of $\bar{t}$ as the supremum of S. Thus, when $y_1(\bar{t}) > 0$ we cannot have $x_0(\bar{t}) > 0$.
- Case 4 ($y_0(\bar{t}) = 0$). The analysis is the same as in Case 3: If $y_1(\bar{t}) = 0$, there is nothing to prove; if $y_1(\bar{t}) > 0$, the definition of $\bar{t}$ as the supremum of S is violated.

In summary: 1) We can never have $x_1(\bar{t}) = 0$. 2) Whenever $y_1(\bar{t}) = 0$, then $z(t) \geq 0$ $\forall t \geq \bar{t}$. 3) If $y_1(\bar{t}) > 0$, neither $x_0(\bar{t})$ nor $y_0(\bar{t})$ can be equal to zero. Therefore, $z(t) \geq 0$ for all $t \geq 0$.

□

Proposition 2. *"The solution $z(t) = (x_1(t), x_0(t), y_1(t), y_0(t))$ of [9] with initial conditions $x_1(0) > 0$, $x_0(0) = 0$, $y_1(0) > 0$ and $y_0(0) = 0$ is non-negative, i.e.: $z(t) \geq 0\ \forall t \geq 0$."*

Proof: Suppose the proposition is false. Then following the proof of Proposition 1, the set $S = \{t \in \mathbb{R}^+ : z(s) > 0 \text{ for } s \in (0, t]\}$ has a least upper bound $\bar{t} > 0$, $z(\bar{t}) \geq 0$, and at least one of the entries of $z(\bar{t})$ must be equal to zero.

- Case 1 ($x_1(\bar{t}) = 0$). If $x_1(\bar{t}) = 0$, then $\dot{x}_1(\bar{t}) = gx_0(\bar{t})$. Hence, we examine two sub cases: (i) $x_0(\bar{t}) > 0$, and (ii) $x_0(\bar{t}) = 0$. Sub case (i): If $x_0(\bar{t}) > 0$, then $x_1(t)$ is strictly increasing in a neighborhood of $\bar{t}$, and thus $\exists\, t^*$ such that $0 < t^* < \bar{t}$ and $x_1(t^*) < x_1(\bar{t}) = 0$, violating the definition of $\bar{t}$ as the supremum of S. We cannot have then $x_1(\bar{t}) = 0$ and $x_0(\bar{t}) > 0$. Sub case (ii): The set $M = \{(x_1, x_0, y_1, y_0) \in \mathbb{R}^4 : x_1 = 0 \text{ and } x_0 = 0\}$ is an invariant manifold of (9). Hence, if $x_1(\bar{t}) = x_0(\bar{t}) = 0$, then $x_1(t) = x_0(t) = 0\ \forall t \geq \bar{t}$. It follows that for $t \geq \bar{t}$, $y_1(t) = y_1(\bar{t})e^{\int_{\bar{t}}^{t} \beta y_0(s) - a\, \mathrm{d}s} \geq 0$ and $y_0(t) = y_0(\bar{t})e^{-\int_{\bar{t}}^{t} a + \beta y_1(s)\, \mathrm{d}s} \geq 0$. In summary, sub case (i) is not possible and sub case (ii) implies that $z(t) \geq 0$ for $t \geq 0$.

- Case 2 ($y_1(\bar{t}) = 0$). Following the proof of Proposition 1 (Case 2), if $y_1(\bar{t}) = 0$, then $y_1(t)$, $y_0(t)$ and $x_0(t)$ are all non-negative for $t \geq \bar{t}$. Next, if $x_1(t) \geq 0\ \forall t \geq \bar{t}$ we have $z(t) \geq 0$ for $t \geq \bar{t}$ and there is nothing left to prove. Suppose there is a $t^* > \bar{t}$ such that $x_1(t^*) < 0$, then the set $R = \{t \geq \bar{t} : x_1(s) \geq 0 \text{ for } s \in [\bar{t}, t]\}$ is bounded and non-empty and has a least upper bound $\hat{t}$. Since x_1 is continuous we must have $x_1(\hat{t}) = 0 \Rightarrow \dot{x}_1(\hat{t}) = gx_0(\hat{t})$. If $x_0(\hat{t}) = 0$, then $\dot{x}_1(\hat{t}) = 0 \Rightarrow x_1(t) = 0 \forall t \geq \hat{t}$, which contradicts the existence of t^*. If $x_0(\hat{t}) > 0 \Rightarrow \dot{x}_1(\hat{t}) > 0$, which means there is a $\delta > 0$ such that $x_1(t)$ is increasing for $t \in [\hat{t}, \hat{t} + \delta]$, which contradicts the definition of $\hat{t}$ as the supremum of R. We must have then $x_1(t) \geq 0\ \forall t \geq \bar{t}$ and it follows that $z(t) \geq 0$ for $t \geq 0$.

- Case 3 ($x_0(\bar{t}) = 0$). If either $x_1(\bar{t})$ or $y_1(\bar{t})$ are equal to zero, then from Cases 1 and 2 it follows that $z(t) \geq 0$ for $t \geq 0$. Furthermore, from the proof of Proposition 1 (Case 3), $x_1(\bar{t})$ and $y_1(\bar{t})$ cannot both be positive when $x_0(\bar{t}) = 0$. It follows that $z(t) \geq 0$ for $t \geq 0$.

- Case 4 ($y_0(\bar{t}) = 0$). The analysis follows the previous arguments. If $y_1(t)$ or $x_0(t)$ are zero, then from Cases 3 and 4, $z(t) \geq 0$ for $t \geq \bar{t}$. If $x_0(\bar{t})y_1(\bar{t}) > 0$, then the definition of $\bar{t}$ as the supremum of S is violated. Hence, $z(t) \geq 0$ for $t \geq 0$.

□

Parameter units

It is valuable here to discuss the units of each of the parameters. First, r, a and d have units of 1/time; K has units of cells; λ has units of cells/time; β has units of 1/(time×cells); and γ has also units of .1/(time×cells)

For comparison purposes we can rescale λ, β, and γ so that every parameter has either units of cells or units of 1/time. Let $L = \lambda/d$ be the equilibrium number of cells prior to infection in models (1),(2), (3), and (4), then $\lambda = Ld$ and if we make $\bar{\lambda} = d$, the rescaled parameter ($\bar{\lambda}$) has units of 1/time. Similarly, if we set $\bar{\beta} = \beta L$ and $\bar{\gamma} = \gamma L$, then the rescaled parameters $\bar{\beta}$ and $\bar{\gamma}$ have also units of 1/time. In the same way for model (S1), (S2), (S3), and (S4) we can set $\bar{\beta} = \beta K$ and

$\bar{\gamma} = \gamma K$. We can then substitute in (1) the parameters λ and β in terms of $\bar{\lambda}$, $\bar{\beta}$ and L. Clearly the models can be rewritten in terms of these rescaled parameters. For example, model (3) becomes:

$$\begin{cases} \dot{x}_1 &= Ld - dx_1 + gx_0 - \left(\frac{\bar{\beta}}{L}\right) x_1 y_1 - \left(\frac{\bar{\gamma}}{L}\right) x_1 y_1 \\ \dot{x}_0 &= \left(\frac{\bar{\gamma}}{L}\right) x_1 y_1 - gx_0 - \left(\frac{\bar{\beta}}{L}\right) x_0 y_1 - dx_0 \\ \dot{y}_1 &= \left(\frac{\bar{\beta}}{L}\right)(x_1 + y_0) y_1 - a y_1 \\ \dot{y}_0 &= \left(\frac{\bar{\beta}}{L}\right) x_0 y_1 - a y_0 - \left(\frac{\bar{\beta}}{L}\right) y_0 y_1 \end{cases} \quad [18]$$

Parameter exploration

We have demonstrated that models (3) and (S3) can have multiply stable steady states depending on the choice of parameters. It is then important to investigate the parameter regions where multiple stability might or might not occur.

Model (3)

First note that the units of time are arbitrary, therefore we can fix the value of one of the parameters and vary the others in relation to that fix parameter. In the simulations we chose to make $\beta = 0.001$. It is also trivial to verify that the stability and number equilibria in system [18] does not depend on the value of L. For the simulations we choose a value of $L = 1000$ (note that we have then $\bar{\beta} = 1$). Next we generated 400,000 random sets of parameters (a, d, γ, g) and color coded them according the number of stable equilibria.[2] Figure E depicts the projections in the six planes determine by the four free parameters. The shape of projection in the $da-$plane is caused by the restriction $d < a$ imposed on the model. Points in yellow represent parameters for which virus extinction was the only stable steady state; points in blue correspond to parameter where the single stable equilibrium included virus persistence; red points correspond to parameters that produce two stable equilibria (bistability); and finally, green points, of which they are very few, correspond to parameters where there was no stable steady state and the system instead displayed oscillatory behavior (see Figure F(i)).

The basic reproductive ratio of the virus $R_0 = \bar{\beta}/a$ is drawn for the da, γa, and $ga-$planes (Figure E). Interestingly R_0 provides some information on the behavior of the system. For example, we did not find a single parameter in the region defined by $R_0 < 1$ where virus persistence occurred. However, a reciprocal strong statement cannot be said about the region defined by $R_0 > 1$. Indeed as this figure shows, all possible outcomes with regards to the persistence or extinction of the virus can occur in the region $R_0 > 1$. (Note that these results correspond to the ODE model and hence the outcomes are not stochastic.)

Model (S3)

To conduct a parameter exploration of model (S3) we again fixed $\beta = 0.001$ and made $K = 1000$. The methods and color coding used was the same as for model (3). However, in this figure we

[2]Note that the classification of the number of stable steady states does not say anything about the domains of attraction of each equilibria.

distinguished an additional outcome related to the extinction of the target cell population. In the ODEs the number of infected cells cannot reach zero, but depending on the parameters, pronounced oscillations in the cell population can drive the number of target cells below one, which in a biological scenario would imply their extinction. For this reason we included a new color code, grey, to represent those parameters (with a single stable equilibria) that resulted in oscillations that pushed the target cell population below one before the infected cell population reached the same threshold, starting from the initial conditions $x_1(0) = K$ and $y_1(0) = 3$ (consistent with our paper's focus on low infection multiplicities). We did not included this option (target cell extinction) in model (3) because the constant rate of production of uninfected cells in [8] does not allow for the extinction of target cells. Figure G presents the results for the parameter exploration, where to limit the number of panels we focused our attention on the case $d = 0$ (no uninfected cell death).

Finally, an interesting observation, is that even though in both models ((3) and (S3)) the number of steady states can number up to five, we did not find any parameter set that produced more than two positive stable equilibria.

Agent-based model

For the stochastic agent-based model we do not have the type of analytical tools that allowed us to classify the parameter sets for models (3) and (S3). For this reason in Figure H we only color code two outcomes: (i) limited infections (yellow), and (ii) robust infections (blue). A point in the parameter space was recorded as a robust infection if in the simulation the infected cell population reached 1000 infected cells. Conversely, points in the parameter space were recorded as limited if in the simulation the infection died out before reaching an infected cell population of 1000 cells. As before we fix the value of β (in this case to $\beta = 1$). Simulations were conducted on large grid of size 1000×1000 cells. In this case the reproductive ratio was defined as $R_0 = \beta k/a$, where $k = 25$ is the size of the local neighborhood of each cell.

Figure Legends

Figure B. (i) Bistability in model (S3). With the same set of parameters, but different initial conditions the trajectories converge to different equilibria. In this panel, if the initial number of infected cells is equal to three ($y_1(0) = 3$), the infection first takes off, but eventually regresses and stabilizes at a very low level (plots labeled with "1" in the figure). If the initial number of infected cells is equal to four the system converges to a persistent and robust infection (plots labeled with "2"). (ii) Bistability in the stochastic mass action model. This panel shows two simulations with identical initial conditions. The simulations results in either a weak, limited infection that is eventually extinguished (labeled with "1"), or in a persistent and robust infection (labeled with "2"). (iii) Distribution of the maximum number of infected cells when there is a limited infection. Although the initial number of infected cells in this panel is very small ($y_1(0) = 3$), at their maximum extension limited infections average approximately 80 infected cells. (iv) Probability of the emergence of a robust infection as a function of the normalized level of the the antiviral induction rate γ. The reduction of the strength of the antiviral induction rate results in an increased probability of establishing a robust infection (1000 simulations per antiviral induction level, error bars represent 95% confidence interval). In panel (i): $r = 10, d = 0, \beta = 8.94 \times 10^{-4}, \gamma =$

1.92×10^{-2}, $g = 4.76 \times 10^{-3}$, $a = 0.136$. In panels (ii), (iii) and (iv): $r = 40$, $d = 0$, $\beta = 1.45 \times 10^{-4}$, $g = 2 \times 10^{-2}$, $a = 2 \times 10^{-2}$. In panel (ii) the value of $\gamma = 2 \times 10^{-2}$. In panel (iii) the value of $\gamma = 2.34 \times 10^{-2}$. In panel (iv) the 100% level of antiviral induction rate corresponds to $\gamma = 2.88 \times 10^{-2}$. In all panels: $x_1(0) = 1000$, and $x_0(0) = y_0(0) = 0$. In panels (ii),(iii), and (iv): $y_1(0) = 3$.

Figure C. (i) Spatial spread of a robust infection in the stochastic meta-population model (S4). There are $n = 51$ local patches of cells. The expected number of cells in each patch prior to the infection is $k = 121$ (indicated by the dashed lines). At the start of the simulation ($t = 0$) all patches contain k uninfected cells and three infected cells are placed in the middle of the spatial array (patch $i = 26$). (ii) Time evolution of the infected and uninfected cell populations for the simulation in panel (i). The populations refer to the total number of cells in the spatial array. (iii) Example of a limited infection in the meta-population model. The infection first takes off, but then stalls and regresses until it is eventually extinguished from the cell population. Parameters: $r = 52.1$, $d = 0$, $\beta = 1.44 \times 10^{-3}$, $\gamma = 0.87$, $g = 2.44 \times 10^{-2}$, $a = 2.18 \times 10^{-2}$, and $m = 0.2$.

Figure D. Stochastic agent-based model. Time series of the number of infected cells for the robust infection (panel i) and limited infection (panel ii) for the simulations in Figure 4(E-G) in the main manuscript. (iii) Distribution of maximum number of infected cells when there is a limited infection. At their maximum extension limited infections average approximately 87 infected cells (based on 4094 simulations). (iv) Probability of the emergence of a robust infection as a function of the normalized level of the the antiviral induction rate for the agent-based model. The reduction of the strength of the antiviral induction rate results in an increased probability of establishing a robust infection (probabilities based on 1000 simulations, error bars represent 95% confidence interval). All parameters and initial conditions are the same as in Figure 4(E-G) in the main manuscript. In (iv) the 100% percent level corresponds to a value of $\gamma = 6.238 \times 10^{3}$.

Figure E. Parameter exploration of model (S3). Based on 400,000 random parameters. The infection rate $\beta = 0.001$, the equilibrium number of uninfected cells prior to infection $L = 1000$. Red: two stable and positive equilibria. Yellow: virus extinction. Blue: virus persistence. Green: no stable positive steady state. Grey: target cell "extinction" (population falls below one). See text for discussion.

Figure F. Examples of non-convergence (oscillatory behavior) in models (8) and (9). (i) Model (8). Parameters: $\lambda = 3.2116$, $\beta = 10^{-3}$, $\gamma = 0.0445$, $g = 0.003$, $a = 0.0487$, $d = 0.0032$. (ii) Model (9). Parameters: $r = 13.4008$, $\beta = 10^{-3}$, $\gamma = 0.9113$, $g = 2.1991$, $a = 0.1487$, $d = 0$, $K = 1000$. Initial conditions for both panels: $x_1(0) = 1000$, $x_0(0) = 0$, $y_1(0) = 6$ and $y_0(0) = 0$.

Figure G. Parameter exploration of model (S4). Based on 400,000 random parameters. The infection rate $\beta = 0.001$, the death rate of uninfected cells $d = 0$, and the carrying capacity $K = 1000$ were held fixed. Red: two stable and positive equilibria. Yellow: virus extinction. Blue: virus persistence. Green: no stable positive steady state. Grey: target cell "extinction" (population falls below one). See text for discussion.

Figure H. Parameter exploration in the 2D stochastic model. Based on 400,000 random parameters (one simulation per parameter). Yellow (limited infection). Blue (robust infections). Grid size

1000×1000, Initial conditions nine infected cells in the center of the grid. Infection was scored as robust if the infected cell population reached 1000 infected cells. If the infected cell population died out before reaching 1000 infected cells, the simulation was scored as limited. $\beta = 1$ in the figure.

References

[Gibson and Bruck, 2000] Gibson, M. A. and Bruck, J. (2000). Efficient exact stochastic simulation of chemical systems with many species and many channels. *The journal of physical chemistry A*, 104(9):1876–1889.

[Gillespie, 1977] Gillespie, D. (1977). Exact stochastic simulation of coupled chemical-reactions. *Journal of Physical Chemistry*, 81(25):2340–2361.

[Nowak and May, 2000] Nowak, M. A. and May, R. M. (2000). *Virus dynamics: mathematical principles of immunology and virology.* Oxford University Press, Oxford.

[Perelson and Ribeiro, 2013] Perelson, A. S. and Ribeiro, R. M. (2013). Modeling the within-host dynamics of hiv infection. *BMC Biol*, 11:96.

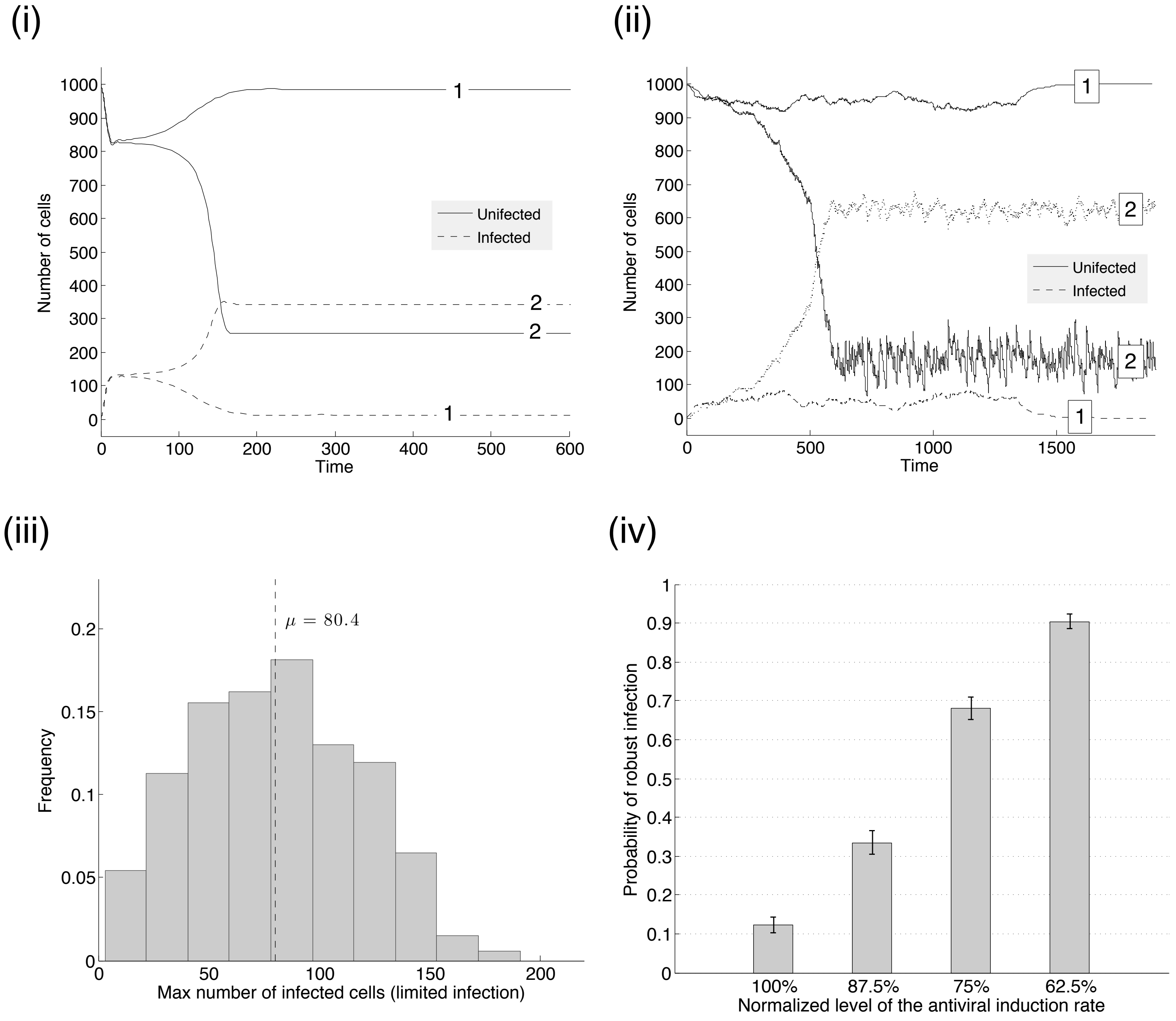
(i)
Number of cells
Time
Unifected
Infected
1
2
(ii)
Number of cells
Time
Unifected
Infected
1
2
(iii)
$\mu = 80.4$
Frequency
Max number of infected cells (limited infection)
(iv)
Probability of robust infection
100%
87.5%
75%
62.5%
Normalized level of the antiviral induction rate

Figure B

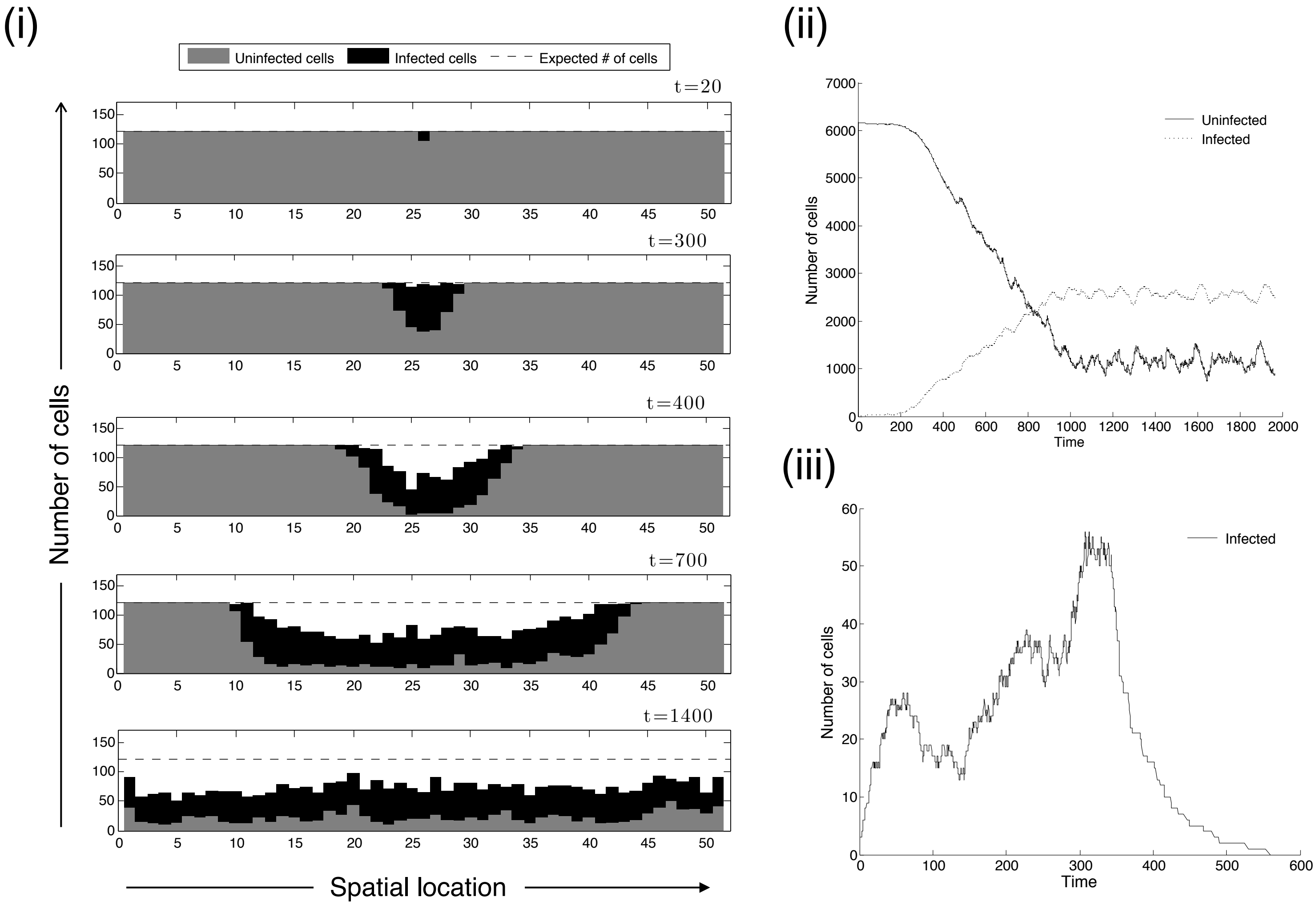

(i)
Uninfected cells
Infected cells
Expected # of cells
t=20
t=300
t=400
t=700
t=1400
Number of cells
Spatial location
(ii)
Uninfected
Infected
Number of cells
Time
(iii)
Infected
Number of cells
Time

Figure C

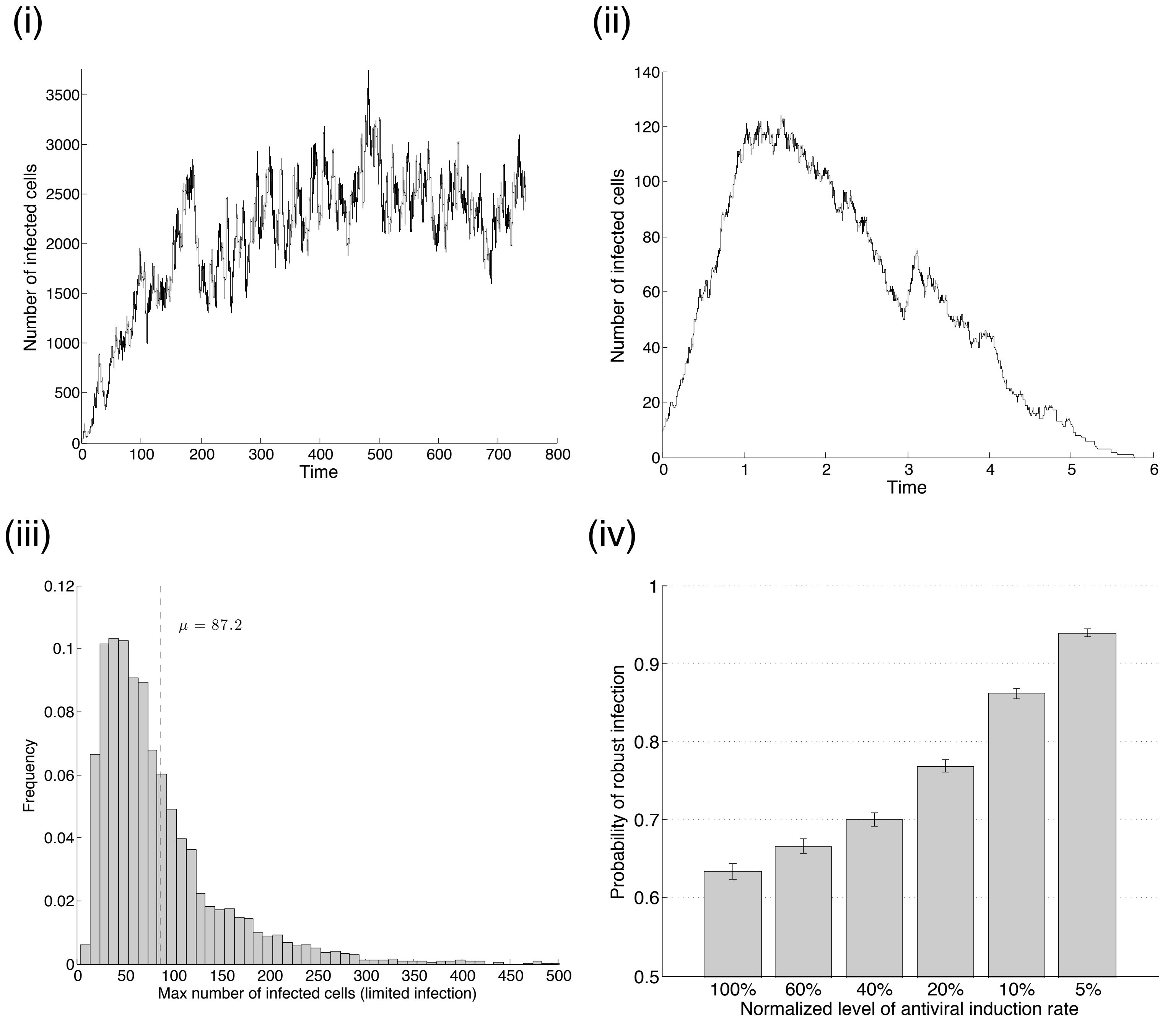
(i)
Number of infected cells
Time
0
500
1000
1500
2000
2500
3000
3500
100
200
300
400
500
600
700
800
(ii)
Number of infected cells
Time
0
20
40
60
80
100
120
140
1
2
3
4
5
6
(iii)
Frequency
Max number of infected cells (limited infection)
$\mu = 87.2$
0
0.02
0.04
0.06
0.08
0.1
0.12
50
100
150
200
250
300
350
400
450
500
(iv)
Probability of robust infection
Normalized level of antiviral induction rate
0.5
0.6
0.7
0.8
0.9
1
100%
60%
40%
20%
10%
5%

Figure D

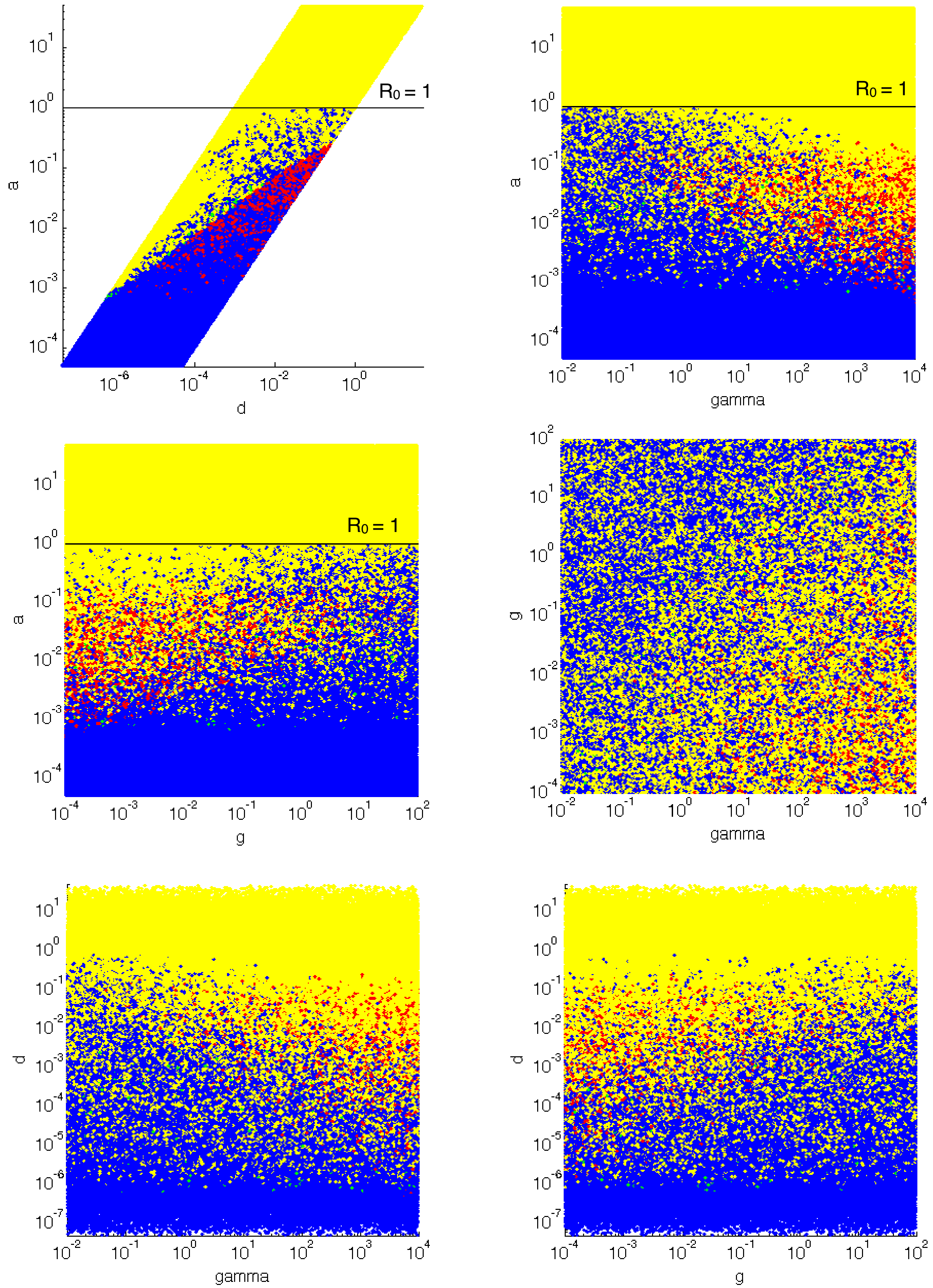

Figure E

(i)

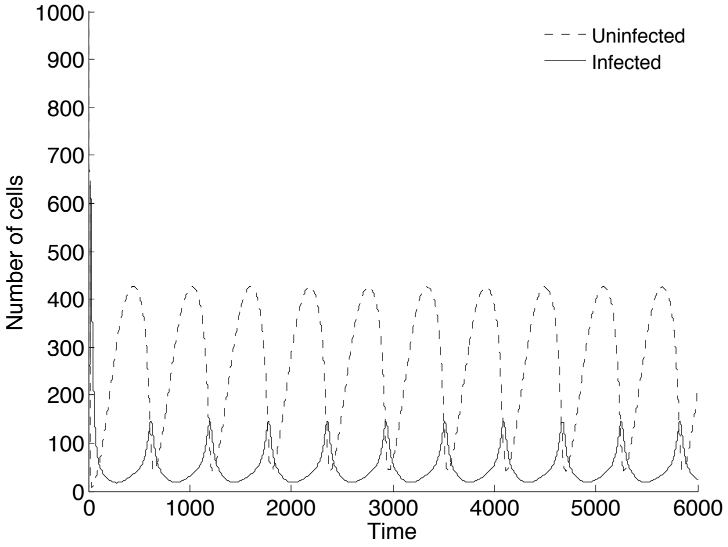

Uninfected
Infected
Number of cells
Time

(ii)

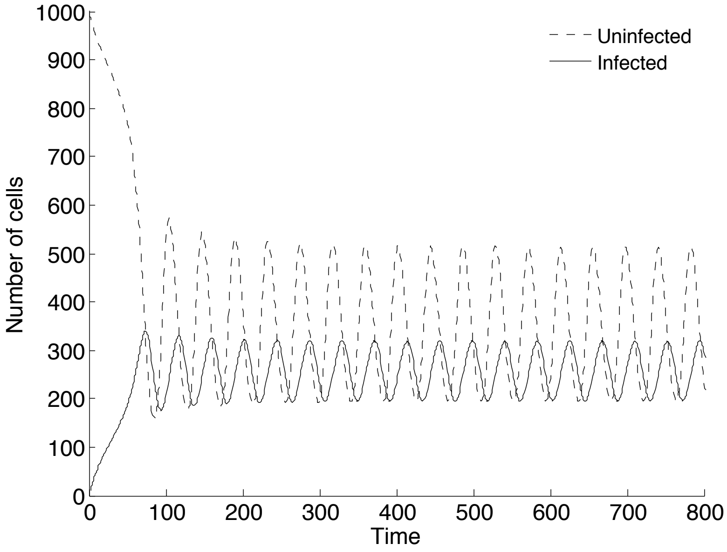

Uninfected
Infected
Number of cells
Time

Figure F

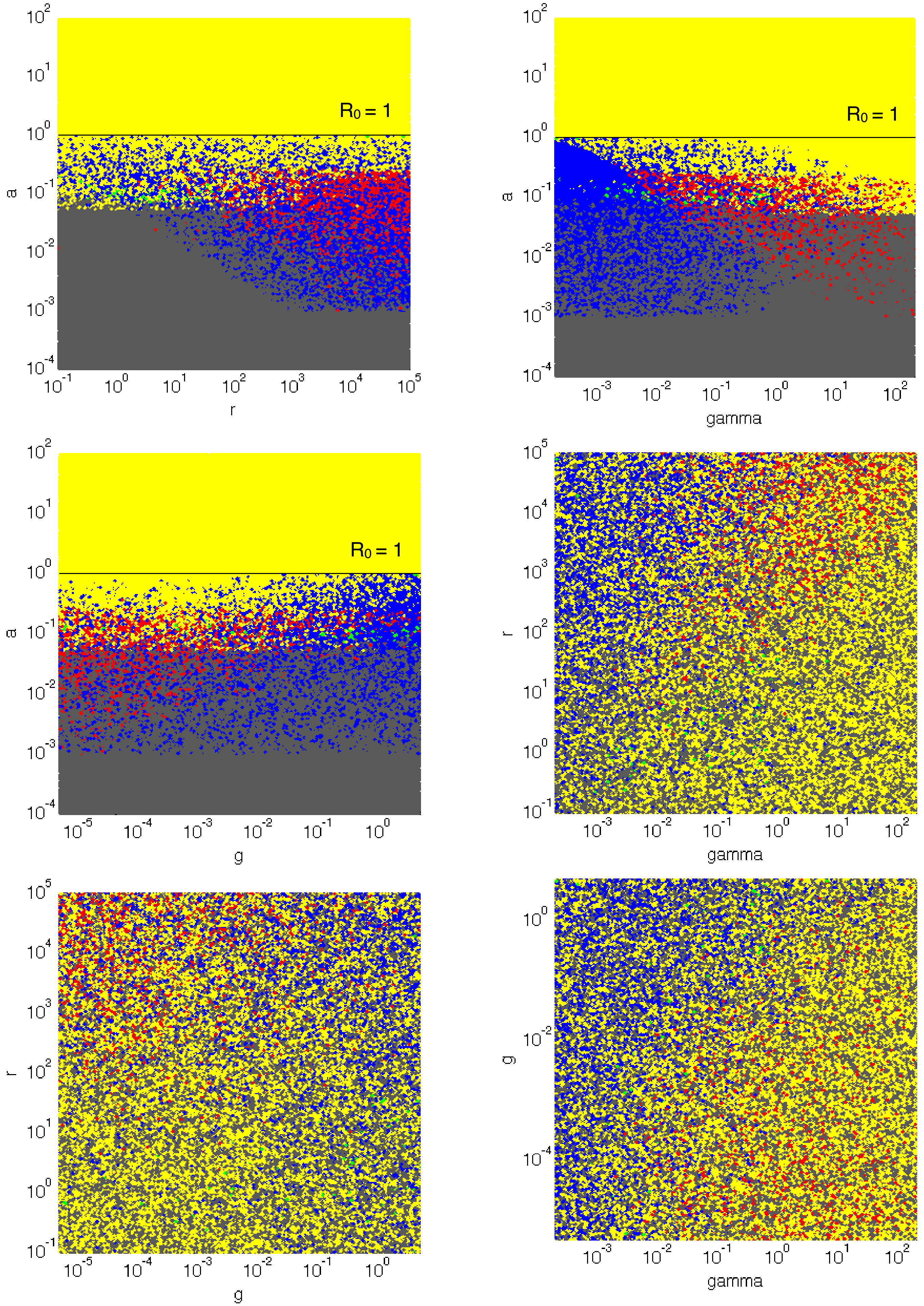
R0 = 1
a
r
gamma
g
10^-1 10^0 10^1 10^2 10^3 10^4 10^5
10^-4 10^-3 10^-2 10^-1 10^0 10^1 10^2
10^-5 10^-4 10^-3 10^-2 10^-1 10^0

Figure G

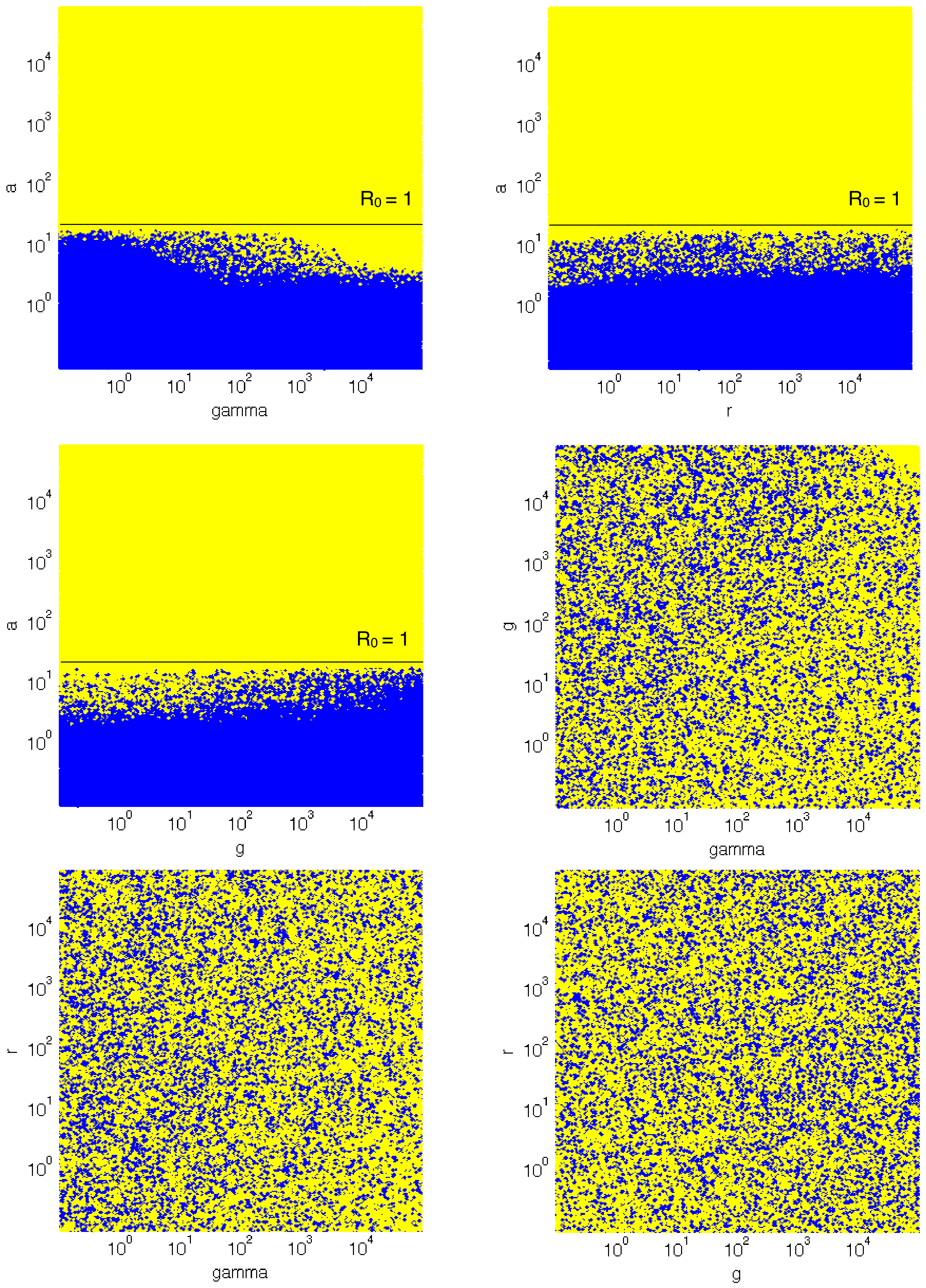

Figure H
